# Using Artificial Intelligence to Predict Intracranial Hypertension in Patients After Traumatic Brain Injury: A Systematic Review

**DOI:** 10.1007/s12028-023-01910-2

**Published:** 2024-01-11

**Authors:** S. T. van Hal, M. van der Jagt, M. E. van Genderen, D. Gommers, J. F. Veenland

**Affiliations:** 1 Department of Adult Intensive Care, Erasmus MC University Medical Center, Room Ne-413, Doctor Molewaterplein 40, Rotterdam, 3015 GD The Netherlands; 2 Department of Medical Informatics, Erasmus MC University Medical Center, Rotterdam, The Netherlands; 3 Department of Radiology, Erasmus MC University Medical Center, Rotterdam, The Netherlands

**Keywords:** Traumatic brain injury, Artificial intelligence, Intracranial hypertension, Prediction

## Abstract

Intracranial hypertension (IH) is a key driver of secondary brain injury in patients with traumatic brain injury. Lowering intracranial pressure (ICP) as soon as IH occurs is important, but a preemptive approach would be more beneficial. We systematically reviewed the artificial intelligence (AI) models, variables, performances, risks of bias, and clinical machine learning (ML) readiness levels of IH prediction models using AI. We conducted a systematic search until 12-03-2023 in three databases. Only studies predicting IH or ICP in patients with traumatic brain injury with a validation of the AI model were included. We extracted type of AI model, prediction variables, model performance, validation type, and prediction window length. Risk of bias was assessed with the Prediction Model Risk of Bias Assessment Tool, and we determined the clinical ML readiness level. Eleven out of 399 nonduplicate publications were included. A gaussian processes model using ICP and mean arterial pressure was most common. The maximum reported area under the receiver operating characteristic curve was 0.94. Four studies conducted external validation, and one study a prospective clinical validation. The prediction window length preceding IH varied between 30 and 60 min. Most studies (73%) had high risk of bias. The highest clinical ML readiness level was 6 of 9, indicating “real-time model testing” stage in one study. Several IH prediction models using AI performed well, were externally validated, and appeared ready to be tested in the clinical workflow (clinical ML readiness level 5 of 9). A Gaussian processes model was most used, and ICP and mean arterial pressure were frequently used variables. However, most studies showed a high risk of bias. Our findings may help position AI for IH prediction on the path to ultimate clinical integration and thereby guide researchers plan and design future studies.

**Supplementary Information:**

The online version contains supplementary material available at 10.1007/s12028-023-01910-2.

## Introduction

Intracranial hypertension (IH) portends a worse prognosis in patients with traumatic brain injury (TBI) and should be treated expediently [1]. The primary brain injury is often accompanied by tissue edema resulting in IH. It is the key driver of secondary brain injury, as it hampers cerebral perfusion and induces ischemia [1]. Current practice has focused mainly on alleviation intracranial pressure (ICP) once it has occurred, by medical or surgical interventions [1]. A preemptive approach may contribute to prevention of ICP surges, by mitigation of contributing factors known to be able to induce secondary brain injury in patients at high risk for IH. Indeed, a rise in ICP reflects exhausted compensatory intracranial reserve (compliance) contributing to secondary injuries that would better be prevented.

Clinical variables contributing to secondary brain injury and IH include fever, hypoosmolality of serum, hyperglycemia, prolonged hyperventilation or hypoventilation, and venous congestion caused by high positive end-expiratory pressure levels of the ventilator and fluid overload [2, 3]. These factors are represented by variables that are continuously monitored at the intensive care unit (ICU) and are highly amenable to treatment. Early identification of the risk of impending IH can enable clinicians or nurses at the bedside to correct or optimize such variables, and thereby decrease the risk of IH, but could also serve as measure to identify patients in need of impending rescue therapies (e.g., decompressive surgery).

To date, prediction of IH with such physiological variables remains understudied.

Contemporary artificial intelligence (AI) algorithms may outperform physicians regarding clinical prediction related to medical conditions [4] or image analysis [5]. In a data driven environment such as the ICU, the potential of AI to aid clinical practice decision making might even be higher [6]. AI is able to analyze vast amounts of data, recognize patterns, and make fast predictions based on these patterns. Hence, AI may be a valuable tool to help early identification of patients at risk of IH and enable earlier treatment to prevent its development.

Although the potential for AI to aid clinical practice may seem high, it is important to assess possible bias, as improper data or model development may result in an AI method that performs well in specific situations, but not in clinical practice. External model validation is crucial to either identify such biased models or endorse the generalizability of a model.

Another important factor for implementation at the bedside is the machine learning (ML) level of readiness [7], which indicates the position of a model on the path from concept to clinical use and improvement of care and outcomes. Determining this level for every study will provide valuable information regarding how “ready” this technology is for real-world applications.

The aim of this systematic review was to assess the available literature regarding the prediction of IH in patients with TBI using validated AI models, and specifically we sought to determine the type of AI methods and variables that are being used to predict IH, the performance of these models, the risk of bias, and the clinical ML readiness level for integration in the clinical workflow.

## Methods

The protocol for this systematic review has been registered in PROSPERO (registration number: CRD42020214744). This research was conducted and reported using the Preferred Reporting Items for Systematic Reviews and Meta-Analyses (PRISMA) [8]. This article adheres to ethical guidelines and did not require ethical approval or use of informed consent.

### Search Strategy

We searched the Embase, Ovid, and Web of Science Core Collection electronic databases on 12-03-2023 for publications describing studies that involved ICP or IH, TBI, and AI, with assistance for electronic search strategies from a medical information specialist. The full queries can be found in Supplementary file 1.

### Inclusion Criteria and Study Selection

Titles and abstracts were screened by two authors (SvH, JV) to assess whether an article was eligible for inclusion. Articles were included if they used AI with the aim to predict ICP/IH in patients with TBI and reported performance measures on an internal, external, or prospective validation set; thus, only articles reporting validated models were included. We excluded non-English articles and nonoriginal literature. Full-text publications were then screened to include all studies that met the inclusion criteria.

### Data Extraction

Data were extracted from the selected studies by two authors (SvH, JV), with focus on the type of AI method used, variables used in the model, area under the receiver operating characteristic curve (AUC), accuracy, sensitivity, and specificity. These concepts are explained in the glossary at the end of this article.

In order to properly assess the risk of bias and the clinical ML readiness level, we also collected the patient inclusion process, type of validation, prediction window length, and the following dataset properties: amount of data instances (explained subsequently) in the training and validation set, the data instance length, percentage of instances preceding IH, sample frequency, IH definition, and data cleaning process.

The term “(data) instance” is used to indicate a collection of data acquired during a certain time period (e.g., 1 h). In retrospective data, it is known whether this collection of data preceded a period of IH, hence a label may be given to the data instance that represents whether an IH event occurred after this period. These instances are subsequently used to train an AI model to recognize distinct patterns that are present in data preceding an IH event and absent in data not preceding an IH event, and vice versa. A properly trained model should subsequently be able to recognize such patterns in real-time data and thus provide predictions.

### Risk of Bias

We analyzed every article using the Prediction Model Risk of Bias Assessment Tool (PROBAST) [9]. We plotted for each domain (participants, predictors, outcomes, and analysis) the risk of bias as percentage of the total number of articles.

### Clinical ML Readiness Level

We assessed the clinical ML readiness level [6, 7], to determine where in the developmental process from concept to clinical integration every model resides. This scoring system indicates how “ready” a model is to use in clinical practice and consists of nine levels: (1) clinical problem identification, (2) proposal of model/solution, (3 and 4) model prototyping & model development, (5) model validation, (6) real-time model testing, (7) workflow implementation, (8) clinical outcome evaluation, (9) and model integration.

### Main Outcome

The main aim of this systematic review is to evaluate and summarize the types of AI methods and variables used, performance measures, risk of bias, and clinical ML readiness levels.

### Statistics

We did not perform a meta-analysis or any statistical analysis because the goal of this review is to provide a qualitative overview of the current literature regarding AI-aided prediction of IH.

## Results

### Study Identification

**Fig. 1:**
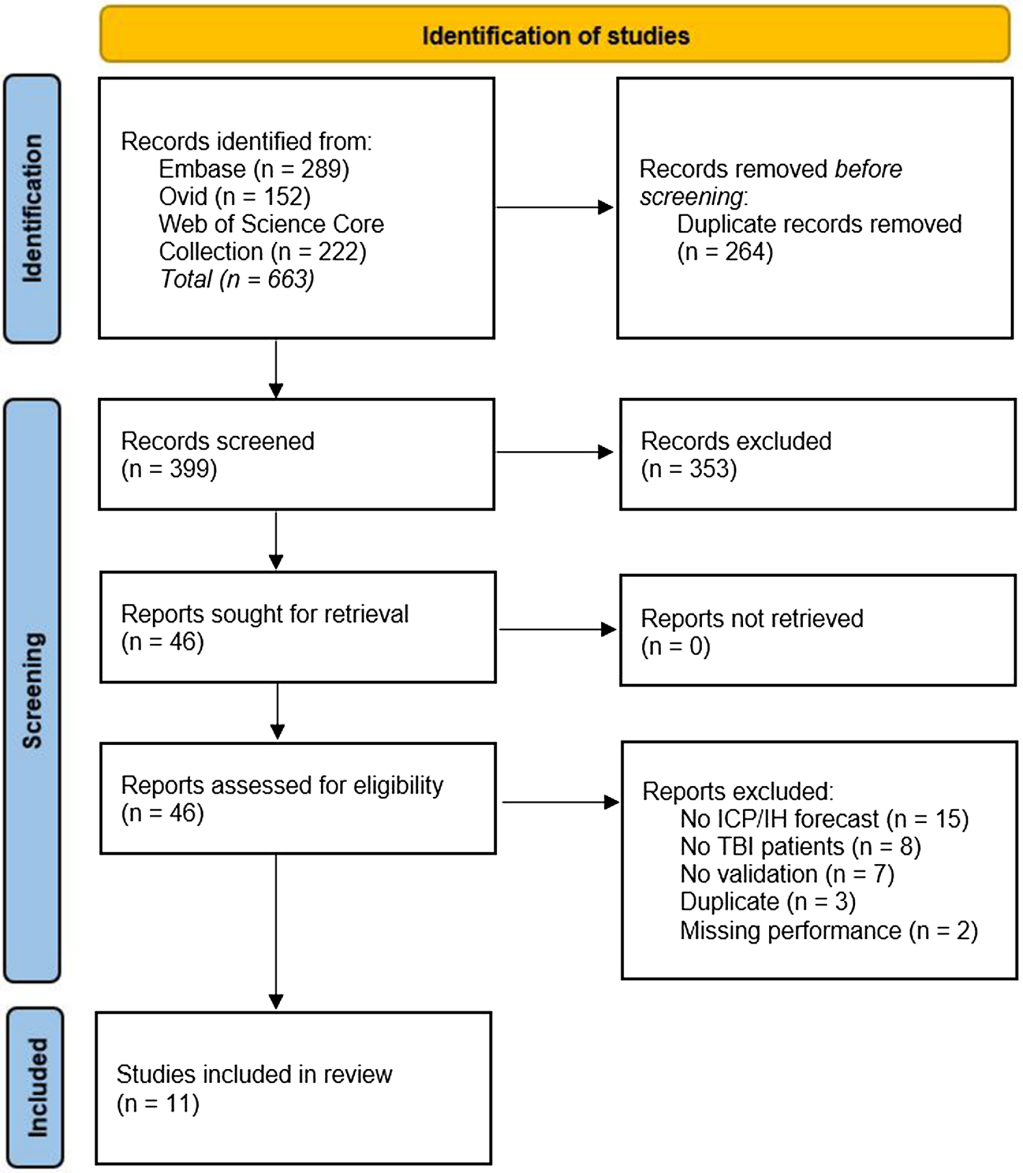
Flowchart of the article selection process. *ICP* intracranial pressure, *IH* intracranial hypertension, *TBI* traumatic brain injury

In total, we identified 399 unique records, of which 11 [10–20] (eight articles, two conference abstracts, and one letter) were eligible for inclusion in this systematic review. A flowchart visualizing the article selection process is provided in Fig. 1. Table 1 summarizes the AI models, clinical variables, study design, and dataset properties.

**Table 1:** Characteristics of each article

Publication	AI methods	Variables	Outcome and prediction window	Sampling	Training data	Validation data	Cleaning
Klauber MR et al. (1984) [10]	LogReg	ICP peak value Abnormal ventricle size on CT Hypotension	IH (ICP > 30 mm Hg)	Presence in first 24 h	156 24-h instances, 55 events (34%)	93 24-h instances, 29 events (31%)	Yes: excluded patients with missing data or IH during first 24 h
			Up to 48 h in advance				
Feng M et al. (2012) [11]	LogReg AODE AdaBoost-J48 BayesNet-K2 BayesNet-TAN LBR naïve Bayes SVM	Intracranial pressure Mean arterial pressure Brain tissue oxygenation Pressure reactivity index	ICP elevation, stability, or reduction	Value per 5 s	1-h instances	1-h instances	Yes: used only data points between interventions
			60 min in advance				
Güiza F et al. (2013) [12]	GP	Intracranial pressure Mean arterial pressure	IH (ICP > 30 mm Hg for 10 min)	Value per 60 s	2,677 4-h instances, 982 events (37%) from 108/178 patients (61%) *(patients with complete records)*	1,135 4-h instances, 392 events (35%) from 33/61 patients (54%)	NP
			30 min in advance				
Beckers M et al. (2014) [13]	GP	Intracranial pressure Mean arterial pressure	IH (ICP > 30 mm Hg for 10 min)	Value per 60 s	Identical to Güiza F et al. (2013) [15]	67 events	NP
			30 min in advance				
Myers RB et al. (2016) [14]	GP LogReg AR-OR	Intracranial pressure Time since last crisis	IH (ICP > 20 mm Hg for 15 min)	Value per 72 s	43,353 30-min instances, 5,979 events (14%) *(patients from 1989–1996)*	38,349 30-min instances, 4025 events (10%) *(patients from 2006–2013)*	Yes: excluded physiologically impossible values, interpolated missing data, used smoothing filter
			30 min in advance				
Güiza F et al. (2017) [15]	GP	Intracranial pressure Mean arterial pressure	IH (ICP > 30 mm Hg for 10 min)	Value per 60 s	Identical to Güiza F et al. (2013) [15]	1051 4-h instances, 231 events (22%) from 41/121 patients (34%)^a^ 2,219 instances, 811 events (37%) from 49/79 patients (62%)^b^	Yes: excluded obvious artifacts
			30 min in advance				
Carra G et al. (2020) [16]	GP	Intracranial pressure Mean arterial pressure	IH (ICP > 30 mm Hg for 10 min)	NP	Identical to Güiza F et al. (2013) [15]	NP	NP
			30 min in advance				
Wijayatunga P et al. (2022) [17]	naïve Bayes	Intracranial pressure	IH (ICP > 20 mm Hg)	Value per 0.008 s	24,108 10-min instances, 4,411 events (18%) *(80% random samples)*	24,108 10-min instances, 4,411 events (18%) *(20% random samples)*	Yes: removed artifacts lasting > 3 s
			Up to 60 min in advance				
Carra G et al. (2022) [18]	GP	Intracranial pressure Mean arterial pressure	IH (ICP > 30 mm Hg for 10 min)	Value per 60 s	Identical to Güiza F et al. (2013) [15]	NP	NP
			30 min in advance				
Petrov D et al. (2023) [19]	RF	Intracranial pressure	IH (ICP > 22 mm Hg for ≥ 75% of 5-min interval)	Value per second	2,795 1-h instances, 656 events (23%) *(model with highest accuracy value)*	NP	Yes: imputed missing data with mean values
			Up to 20 min in advance				
Carra G et al. (2023) [20]	RF using multiple GP	Intracranial pressure Mean arterial pressure	IH (ICP > 15 mm Hg for 180 min up to ICP > 34 mm Hg for 10 min)	Value per 60 s	20,938 4-h instances	25,261 instances, 8,421 events (50%)	Yes: imputed missing data with mean values
			30 min in advance				

This table summarizes for each publication the used forecasting models and variables, the model outcome (i.e., ICP or IH as forecasting outcome, including IH threshold, and the prediction window), the used sample frequency, specifics regarding the data used to train and validate the models, and whether or not some sort of data cleaning has been performed, including a brief description. Of note, five studies [12, 13, 12–13, 18] used the same model based on the same variables

*AdaBoost-J48* ada-boosting with decision tree, *AI* artificial intelligence, *AODE* aggregating one-dependence estimators, *AR-OR* autoregressive ordinal-regression, *BayesNet-K2* Bayesian network with K2, *BayesNet-TAN* Bayesian network with TAN, *GP* Gaussian processes, *ICP* intracranial pressure, *IH* intracranial hypertension, *LBR* lazy Bayesian rules, *LogReg* logistic regression, *NA* not applicable, *naïve Bayes* naïve Bayesian classifier, *NP* not provided, *RF* random forest, *SVM* support vector machine

^a^Adult cohort

^b^Pediatric cohort

### AI Models and Clinical Variables

Eleven different model types were used. Güiza et al. [12], Beckers et al. [13], Güiza et al. [15], Carra et al. [16] and Carra et al. [18] used the same Gaussian processes (GP) model based on ICP and mean arterial pressure (MAP), on different datasets. Feng et al. [11] used eight different models. GP was the most commonly used model, followed by logistic regression and random forest.

To predict ICP/IH, all articles used preceding ICP, and seven [11–13, 11–13, 18, 20] of 11 studies also used MAP. In addition to these two variables, one study [11] also used the brain tissue oxygenation and pressure reactivity index. Another study [10] used the presence of abnormal ventricle size on computed tomography (CT) and hypotension (defined as systolic blood pressure less than 90 mm Hg) within the first 24 h of admission, besides the highest preceding ICP value. One study [14] used only the ICP and the time since last IH event.

### Study Design and Data Set Properties

Six [10–12, 14, 17, 19] studies performed internal validation and four [13, 15, 16, 20] performed external validation (Table 2). We found one report [18] on AI-based IH prediction being tested in clinical practice for patient care.

**Table 2:** Study characteristics

Publication	Validation	ML readiness level	PROBAST score
Klauber MR et al. (1984) [10]	Internal	3 and 4	High
Feng M et al. (2012) [11]	Internal	3 and 4	High
Güiza F et al. (2013) [12]	Internal	3 and 4	Low
Beckers M et al. (2014) [13]	External	5	High
Myers RB et al. (2016) [14]	Internal	3 and 4	High
Güiza F et al. (2017) [15]	External	5	High
Carra G et al. (2020) [16]	External	5	High
Wijayatunga P et al. (2022) [17]	Internal	3 and 4	High
Carra G et al. (2022) [18]	Prospective	6	Low
Petrov D et al. (2023) [19]	Internal	3 and 4	High
Carra G et al. (2023) [20]	External	5	Low

This table summarizes for each publication the validation type, machine learning (ML) readiness level, and Prediction Model Risk of Bias Assessment Tool (PROBAST) score

All studies investigated the prediction of IH specifically, except for one [11] study that looked at the ICP course independent of a specific threshold. We found six different definitions of IH: one study [20] defined nine different IH thresholds, ranging from ICP > 15 mm Hg for 180 min up to ICP > 34 mm Hg for 10 min, whereas others used a single threshold of ICP > 30 mm Hg for 10 min [12, 13, 12–13, 18], ICP > 30 mm Hg [10], ICP > 20 mm Hg for 15 min [14], ICP > 22 mm Hg for ≥ 75% of a 5-min interval [19], or ICP > 20 mm Hg [17].

The prediction windows varied from 10 min [17] to 48 h [10], and for 82% of the articles it ranged between 30 and 60 min.

Data instance length ranged from 10 min [17] to 24 h [10]. Data samples were taken every 0.008 s [17] up to one value per 24 h [10].

### Performance

**Fig. 2:**
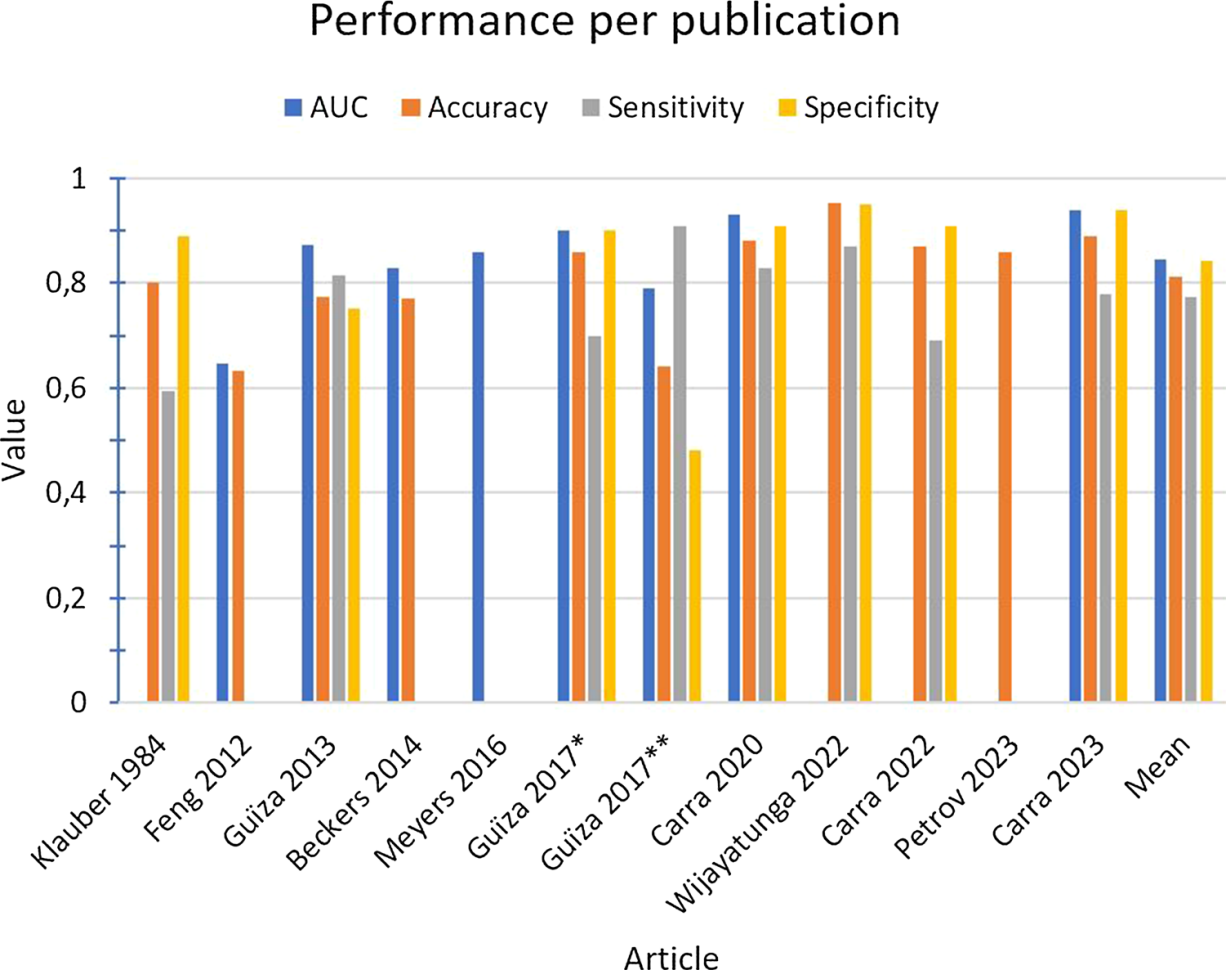
Performance measures of the included articles. *AUC* area under the receiver operating characteristic curve. *Adult cohort. **Pediatric cohort

Table 3 shows the AUC, accuracy, sensitivity, and specificity for each study if reported, and additionally summarizes these for the five [12, 13, 12–13, 18] studies that used the same model. These performance measures are also visualized in Fig. 2. Solely the articles by Güiza et al. [15] and Myers et al. [14] provided 95% confidence intervals. Seven [11–16, 20] publications provided the AUC, ranging from 0.647 [11] to 0.94 [20], with an average of 0.85. All publications but one [14] mentioned an accuracy value, ranging from 63.3 [11] to 95.3% [17], with an average of 81%. Seven [10, 12, 15–18, 20] articles also reported the sensitivity and specificity values, ranging from 59.3 [10] to 91% [15] (with an average of 77%) and from 48 [15] to 95% [17] (with an average of 84%), respectively.

**Table 3:** Model performances, including summary of identical models

Publication	AI methods	AUC	Accuracy	Sensitivity	Specificity
Klauber MR et al. (1984) [10]	LogReg	NP	80.2%	59.3%	89.1%
Feng M et al. (2012) [11]	LogReg AODE AdaBoost-J48 BayesNet-K2 BayesNet-TAN LBR naïve Bayes SVM Best^a^	0.645 0.66 0.632 0.648 0.644 0.647 0.638 0.613 *0.647*	62.1% 62.4% 61.5% 62.3% 62.0% 63.3% 61.9% 62.4% *63.3%*	NP NP NP NP NP NP NP NP *NA*	NP NP NP NP NP NP NP NP *NA*
Güiza F et al. (2013) [12]	GP	0.872	77.4%	81.6%	75.2%
Beckers M et al. (2014) [13]	GP	0.83	77%	NP	NP
Myers RB et al. (2016) [14]	GP LogReg AR-OR	NP NP 0.86 (0.85–0.86)	NP NP NP	NP NP NP	NP NP NP
Güiza F et al. (2017) [15]	GP GP	0.90 (0.87–0.91)^b^ 0.79 (0.77–0.81)^c^	86% (84–88)^b^ 64% (62–66)^c^	70% (64–76)^b^ 91% (90–93)^c^	90% (88–92)^b^ 48% (45–51)^c^
Carra G et al. (2020) [16]	GP	0.93	88%	83%	91%
Wijayatunga P et al. (2022) [17]	naïve Bayes	NP	95.3%^d^	87.1%^d^	95%^d^
Carra G et al. (2022) [18]	GP	NP	87%	69%	91%
Petrov D et al. (2023) [19]	RF	NP	86%	NP	NP
Carra G et al. (2023) [20]	RF using multiple GP	0.94	89%	78%	94%
*Average*		*0.85*	*81%*	*77%*	*84%*
Original model					
Güiza F et al. (2013) [12]	GP	0.872	77.4%	81.6%	75.2%
External validation studies of original model					
Beckers M et al. (2014) [13]	GP	0.83	77%	NP	NP
Güiza F et al. (2017) [15]	GP GP	0.90 (0.87–0.91)^b^ 0.79 (0.77–0.81)^c^	86% (84–88)^b^ 64% (62–66)^c^	70% (64–76)^b^ 91% (90–93)^c^	90% (88–92)^b^ 48% (45–51)^c^
Carra G et al. (2020) [16]	GP	0.93	88%	83%	91%
Carra G et al. (2022) [18]	GP	N.P	87%	69%	91%
*Average*		*0.86*	*80.4%*	*78%*	*80%*

This table summarizes for each publication the performance measures. This table also summarizes the performance measures of the five studies that used the same model. The 95% confidence intervals are provided between brackets if they were reported

*AdaBoost-J48* ada-boosting with decision tree, *AI* artificial intelligence, *AODE* aggregating one-dependence estimators, *AR-OR* autoregressive ordinal-regression, *AUC* area under the receiver operating characteristic curve, *BayesNet-K2* Bayesian network with K2, *BayesNet-TAN* Bayesian network with TAN, *GP* Gaussian processes, *LBR* lazy Bayesian rules, *LogReg* logistic regression, *NA* not applicable, *naïve Bayes* naïve Bayesian classifier, *NP* not provided, *RF* random forest, *SVM* support vector machine

^a^Performance of model with highest accuracy value

^b^Adult cohort

^c^Pediatric cohort

^d^Performance of model with lowest overall error rate

Data cleaning was described by seven studies [10, 11, 10–11, 17, 10–11] and consisted of imputing missing data points with mean values [19, 20], removing values registered during an intervention [11], removing obvious artifacts [15, 17], excluding patients with missing data [10], excluding samples containing IH [10], excluding physiologically impossible values [14], interpolating missing data points [14], and using a smoothing filter [14].

Seven [12, 14–17, 14–17] out of 11 studies mentioned (partial) demographics of their cohort. Of note, Güiza et al. [15] studied an adult and a pediatric cohort. The demographic features that were reported are summarized in Table 4. The lowest and highest age in the reported interquartile ranges were 7.5 [15] and 80 [17], respectively. The percentage of male participants ranged from 74 [15] to 87% [14]. For every study population, the median total Glasgow Coma Score (GCS) was 6 or 7, excluding the studies by Myers et al. [14], that only reported the eye and motor components of the GCS, and by Petrov et al. [19], that only mentioned a GCS of < 8.

**Table 4:** Patient demographics of the training cohorts and validation cohorts

Publication	Patients in data set	Age in years, median [IQR] or mean (range)	Male sex	GCS score, median (IQR)
Klauber MR et al. (1984) [10]	Training: 156	NP	NP	NP
	Validation: 93	NP	NP	NP
Feng M et al. (2012) [11]	Training: 82^a^	NP	NP	NP
	Validation: 82^a^	NP	NP	NP
Güiza F et al. (2013) [12]	Training: 178	33.1 (19–49)	80.9%	7 (4–10)
	Validation: 61	24 (13–44)	77.1%	7 (4–9)
Beckers M et al. (2014) [13]	Training: NA	NA	NA	NA
	Validation: 43	NP	NP	NP
Myers RB et al. (2016) [14]	Training: 368	29 (21–40)	87%	7 (4–9)^b^, 5 (2–5)^c^
	Validation: 261	30 (23–46)	85%	7 (3–8)^b^, 5 (2–5)^c^
Güiza F et al. (2017) [15]	Training: NA^d^ Training: NA^e^	NA^d^ NA^e^	NA^d^ NA^e^	NA^d^ NA^e^
	Validation: 121^d^ Validation: 79^e^	50 (28.5–65)^d^ 10.4 (7.5–14.2)^e^	78%^d^ 74%^e^	7 (3–12)^d^ 6 (5–8)^e^
Carra G et al. (2020) [16]	Training: NA	NA	NA	NA
	Validation: 257	47 (30–61)	81%	6 (3–10)
Wijayatunga P et al. (2022) [17]	Training: 29	56 [20–80]	76%	NP
	Validation: 1	56 [20–80]	76%	NP
Carra G et al. (2022) [18]	Training: NA	NA	NA	NA
	Validation: 14	NP	NP	NP
Petrov D et al. (2023) [19]	Training: 30	NP	NP	< 8
	Validation: 5	NP	NP	< 8
Carra G et al. (2023) [20]	Training: 290	42 (27–56)	80	7 (4–11)
	Validation: 264	47 (29–61)	81	6 (3–10)

*GCS* Glasgow Coma Scale, *IQR* interquartile range, *NA* not applicable, *NP* not provided

^a^Same patients used for training and validation

^b^Eye score

^c^Motor score

^d^Adult cohort

^e^Pediatric cohort

None of the included studies reported that their data or models were publicly available.

### Risk of Bias

**Fig. 3:**
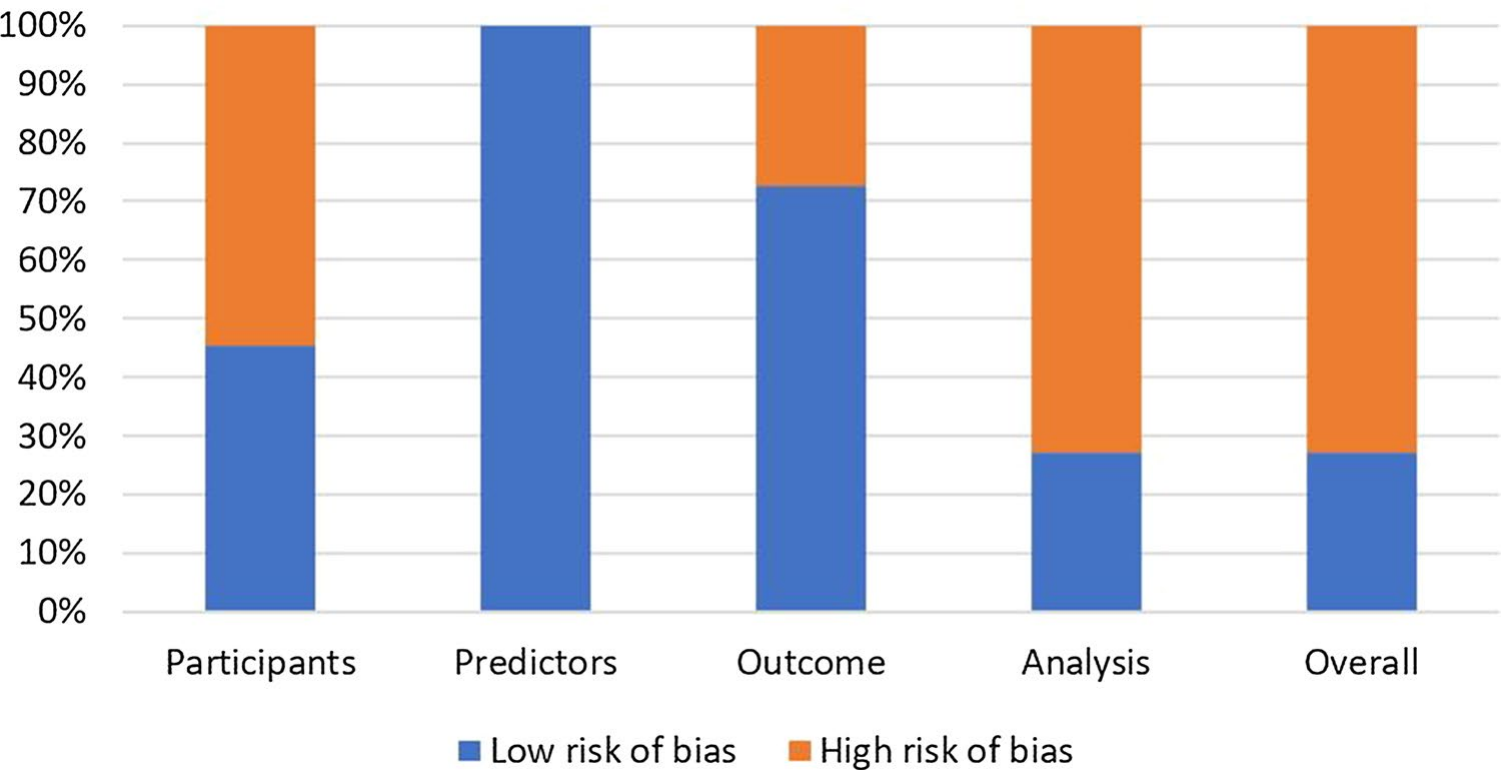
Risk of bias of the included articles, for each PROBAST domain. *PROBAST* Prediction model Risk of Bias Assessment Tool

The results of the PROBAST assessment can be found in Table 2 and are visualized in Fig. 3. The overall risk of bias was found to be high in all but three articles [12, 18, 20], mainly caused by the participants and analysis domains. The full PROBAST assessments are provided in Supplementary file 2.

### Clinical ML Readiness Level

The clinical ML readiness level of the models (Table 2) was at least level 3 and 4 (five out of 11 studies) and at most level 6 (one out of 11 studies).

## Discussion

In this systematic review on AI algorithms to predict ICP/IH in patients with TBI, we found that GP was the most commonly used model, followed by logistic regression and random forest. Only limited variables were used (mainly ICP and MAP). Validated models perform well, with the best AUC being 0.94. Most studies (73%) were classified as having a high risk of bias. The clinical ML readiness level was level 6 for one [18] study, and level 5 at most for all other studies, implying that most models have not left the validation phase and have yet to be tested in clinical practice. Still, these findings indicate that AI-aided prediction of IH in patients with TBI has a very good predictive potential and appears ready for the next steps to achieve clinical integration.

The validated models included in this study perform well and seem robust enough to be subsequently tested in real-time, representing clinical ML readiness level 6. This means that theoretically they appear ready for testing after integration in clinical patient data management systems with the aim to establish their real-life performance, but not yet with the aim to test usability/feasibility (clinical ML readiness level 7), performing phase 3 randomized clinical trials (clinical ML readiness level 8) or to be actually used for and integrated into patient management (clinical ML readiness level 9).

The random forest model by Carra et al. [20], based on the ICP and MAP, achieved the best AUC (0.94) when used on an external validation data set. Of note, the model by Güiza et al. [12] achieved a very similar AUC of 0.93. Although the model used in that study was developed on patient data from 2003 to 2005 (AUC 0.872) [12] and was initially only internally validated, it still accomplished good results when validated externally on data from the AVERT-IT database (AUC 0.83) [13], data from 2009 to 2013 (AUC 0.90 [adult cohort] and 0.79 [pediatric cohort]) [15] and 2015 to 2017 (AUC 0.93) [16], indicating the robustness of this model.

Of note, the AI algorithms mainly used pressure-related variables, with some studies also using brain tissue oxygenation [11], abnormal ventricle size on CT [10], and the time since last IH event [14]. Conspicuously, the most used variables (ICP and MAP) are the determinants for the assessment of cerebral autoregulation with the pressure reactivity index, and therefore the findings of this study may indicate that early changes in cerebral autoregulation predict IH, which from a pathophysiological point of view, is understandable.

Of the included publications, 73% was judged to be high risk of bias. We found a similar pattern regarding the risk of bias per individual domain as Van de Sande et al. [6], who studied AI applications in general critical care. That is, mainly the participants and the analysis domain were classified as high risk. This may be improved by avoiding exclusion of patients and their data as much as possible, in order to mimic clinical practice and build large datasets.

Furthermore, we found that the clinical ML readiness level of only one model [18] was level 6 (real-time model testing in clinical practice), but this concerned an abstract publication. In contrast, the other models that were published did not exceed level 5, i.e., external validation, preceding the first steps toward clinical real-time testing. Thus, this next step of prospective clinical assessment (corresponding to clinical ML readiness level 6) is required in order to reach the next levels [7].

In a recent article, McNamara et al. [21] provided a narrative review including an in-depth theoretical and technical discussion of various ICP forecasting methods and IH prediction algorithms. In our current article, we further built on the review by McNamara et al. [21] in several distinct ways: (1) we performed a systematic rather than a nonsystematic review following the PRISMA guidelines; (2) we only included validated AI models; (3) we assessed the risk of bias using the PROBAST guidelines; (4) we focused on establishing the positioning of the current status of AI prediction models within the framework of clinical ML readiness levels as proposed by Fleuren et al. [7], with the aim to inform the clinical and scientific community on further concrete steps on the pathway toward clinical integration, as described in the second paragraph of this discussion.

The limitations of this study are that the most robust results to date come from several studies from the same research group [12, 13, 12–13, 18], which may hamper generalizability of the findings, despite reported external validations. Seven [12, 14–17, 14–17] out of 11 included studies (partially) described their population demographics. No study explicitly stated that all patient data from a specific time period were used, so we were unable to rule out cherry picking of patient data. Selection bias could therefore be a concern, because selecting patients without missing data or artifacts and with very evident trends in the data may lead to flattering results, whereas using real-world data might yield different prediction properties. Furthermore, no articles stated exactly how many data instances were sampled per patient, which hampers comparability of studies and insight into data collections underlying the AI algorithms. Moreover, the included studies differed regarding the definition of IH, the used sample frequency and the used data instance length. Finally, the lack of external validation of five models [10, 11, 14, 17, 19] reduces the generalizability of their reported results.

Several considerations can be made regarding future research and next steps, based on our findings. First, mainly the ICP and MAP, which are pressure-related features, are used as variables in the included studies. It may be useful to also take other homeostasis-related variables into account, such as serum osmolality or blood glucose levels, to try and improve prediction. Importantly, these variables can be mitigated by clinical treatment, making them interesting from a therapeutic perspective. Second, the use of imaging (especially CT-scan) features in the prediction of ICP/IH in patients with TBI holds promise in relation to AI. Only one study [10] used an imaging-related feature; the presence of abnormal ventricle size on CT. Future studies should explore the use of imaging features to train a predicting method, since AI may especially be able to outperform human interpretation [22]. Third, although the required sample frequency was a value per minute for the best performing model by Carra et al. [20], another recent large validation study by Schweingruber et al. [23] that was excluded in this systematic review given that the minority (less than a third) of included patients with brain injury were actually TBI, found that hourly sampling and missing data could still result in a high AUC (0.94–0.98 with 1 h prediction window). Using a higher sample frequency results in many data points and may be challenging to analyze, and therefore using lower frequency sampling is appealing when predictive properties could be maintained. Fourth, the training data instances varied from 10 min [17] to 24 h [10]. The necessary data instance length likely depends on the type of model. More importantly, prediction window length (the time between a data instance and the actual IH event) will need to be guided by clinical reasoning: which prediction time window will be required to perform timely interventions that may effectively and durably prevent IH and/or cerebral edema and/or progressive traumatic intracranial hemorrhage? Ultimately, intervention studies aimed at improving clinical outcomes will be able to provide the answer, but until then, we should base this on clinical reasoning alone. Whether a time window is appropriate in a particular case will also be influenced by the underlying pathophysiology. The validation study by Schweingruber et al. [23], mainly in patients without TBI, indicated that longer prediction windows up to 24 h appeared possible, albeit with a somewhat lower AUC of 0.78–0.83 compared with shorter prediction windows of 1 h. Fifth, whether the clinical ML readiness level is such that prospective clinical studies should be designed based on prediction windows of up to 1 h, or that further research should first be done in the lower readiness levels of up to 5 (external validation studies) to evaluate models with longer prediction windows, is uncertain at this time since it is as yet uncertain whether preventive interventions within 1 h of IH are as effective as interventions beyond that window. A possible barrier that should also be taken into account is whether clinicians could be persuaded that preventive actions could be effective to abolish IH from occurring later. For instance, in spite of recent evidence from a large prospective multicenter study, showing that careful ICU management including avoiding positive fluid balances [3] in patients with TBI appears beneficial, embracing actions to adapt clinical practice and uptake of research findings may lag behind.

In the context of managing ICP/IH, it is crucial to consider the delicate balance between treatment intensity, the risk of escalating therapies and their potential complications against the expected harm from IH. Balancing treatment intensity and risk against expected harm is an ongoing and dynamic process. When AI tools are thoroughly validated in the future, these tools can support clinicians in their decision-making process by providing predictive capabilities.

## Conclusions

Artificial intelligence–aided prediction of IH in patients with TBI is not yet ready for clinical integration, although predictive properties are good, and the number of external validation studies is growing.

Some well-performing models have been developed, mainly GP using ICP and MAP, with performance up to an AUC of 0.94. There is potential for improvement regarding the risk of bias, and there is a lack of translation of these models toward clinical application. Based on these findings, we summarized and discussed steps that may contribute to eventual integration of these models into the clinical workflow of treating patients with TBI based on the recently proposed framework of clinical ML readiness levels.

### Supplementary Information

Below is the link to the electronic supplementary material.

Supplementary file1 (DOCX 14 kb)

Supplementary file2 (DOCX 96 kb)
