# Supplementary material for: Using Artificial Intelligence to Predict Intracranial Hypertension in Patients After Traumatic Brain Injury: A Systematic Review
Source: Neurocrit Care. 2024 Jan 11;41(1):285–96. doi: 10.1007/s12028-023-01910-2 (PMC11335950; doi:10.1007/s12028-023-01910-2)
Supplement: Supplementary file 1 — Supplementary file1 (DOCX 14 kb) [file 12028_2023_1910_MOESM1_ESM.docx]

Supplementary table 1: Search queries

| **Database** | **Query** |
| --- | --- |
| Embase | ('intracranial hypertension'/de OR 'intracranial pressure monitoring device'/de OR 'intracranial pressure monitoring'/de OR 'intracranial pressure'/de OR (((intracranial* OR cranial*) NEAR/3 (hypertens* OR pressure*))):ab,ti) AND ('traumatic brain injury'/exp OR 'brain injury'/de OR 'head injury'/de OR (((brain OR cereb* OR contusion* OR head* OR cranial* OR cranium*) NEAR/3 (trauma* OR posttrauma* OR injur* OR blunt OR penetrat*)) OR tbi):ab,ti) AND ('machine learning'/exp OR 'artificial intelligence'/exp OR 'predictive model'/de OR (prediction/de AND algorithm/de) OR ((prediction/de OR prognosis/de OR automation/de OR algorithm/de OR 'predictor variable'/de OR 'predictive value'/de) AND (model/de)) OR ((machine NEAR/3 learning) OR (vector NEAR/3 machine) OR (Markov NEAR/3 model) OR (feature NEAR/3 (detection OR extraction OR learning OR ranking OR selection)) OR Bayesian OR (algorithm* NEAR/3 predict*) OR ((network) NEAR/3 learning) OR artificial-neural-network* OR automated-pattern-recognition* OR classification-algorithm* OR classifier* OR computer-heuristics* OR cross-validation* OR data-mining* OR fuzzy-system* OR iterative-closest-point* OR k-nearest-neighbor* OR kernel-method* OR knowledge-discovery* OR memristor* OR molecular-docking* OR multicriteria-decision-analysis* OR multifactor-dimensionality-reduction* OR online-analytical-processing* OR outlier-detection* OR perceptron* OR radial-basis-function* OR random-forest* OR recursive-feature-elimination* OR recursive-partitioning* OR rough-set* OR convolution*-neur*-network* OR ((ambient OR artificial*) NEAR/3 intelligen*) OR (automat* NEAR/3 reasoning*) OR (Computer NEAR/3 heuristic*) OR Multicriteria-decision-analysis OR ((predict* OR forecast* OR prognos*) NEAR/3 (model* OR automat* OR algorit*))):Ab,ti) |
| Ovid | (Intracranial Hypertension/ OR Intracranial Pressure/ OR (((intracranial* OR cranial*) ADJ3 (hypertens* OR pressure*))).ab,ti.) AND (Brain Injuries, Traumatic/ OR Brain Injuries/ OR Craniocerebral Trauma/ OR (((brain OR cereb* OR contusion* OR head* OR cranial* OR cranium*) ADJ3 (trauma* OR posttrauma* OR injur* OR blunt OR penetrat*)) OR tbi).ab,ti.) AND (exp Artificial Intelligence/ OR ((Prognosis/ OR Automation/ OR Algorithms/ OR Predictive Value of Tests/) AND (Models, Theoretical/)) OR ((machine ADJ3 learning) OR (vector ADJ3 machine) OR (Markov ADJ3 model) OR (feature ADJ3 (detection OR extraction OR learning OR ranking OR selection)) OR Bayesian OR (algorithm* ADJ3 predict*) OR ((network) ADJ3 learning) OR artificial-neural-network* OR automated-pattern-recognition* OR classification-algorithm* OR classifier* OR computer-heuristics* OR cross-validation* OR data-mining* OR fuzzy-system* OR iterative-closest-point* OR k-nearest-neighbor* OR kernel-method* OR knowledge-discovery* OR memristor* OR molecular-docking* OR multicriteria-decision-analysis* OR multifactor-dimensionality-reduction* OR online-analytical-processing* OR outlier-detection* OR perceptron* OR radial-basis-function* OR random-forest* OR recursive-feature-elimination* OR recursive-partitioning* OR rough-set* OR convolution*-neur*-network* OR ((ambient OR artificial*) ADJ3 intelligen*) OR (automat* ADJ3 reasoning*) OR (Computer ADJ3 heuristic*) OR Multicriteria-decision-analysis OR ((predict* OR forecast* OR prognos*) ADJ3 (model* OR automat* OR algorit*))).ab,ti.) |
| Web of Science Core Collection | TS=(((((intracranial* OR cranial*) NEAR/2 (hypertens* OR pressure*)))) AND ((((brain OR cereb* OR contusion* OR head* OR cranial* OR cranium*) NEAR/2 (trauma* OR posttrauma* OR injur* OR blunt OR penetrat*)) OR tbi)) AND (((machine NEAR/2 learning) OR (vector NEAR/2 machine) OR (Markov NEAR/2 model) OR (feature NEAR/2 (detection OR extraction OR learning OR ranking OR selection)) OR Bayesian OR (algorithm* NEAR/2 predict*) OR ((network) NEAR/2 learning) OR artificial-neural-network* OR automated-pattern-recognition* OR classification-algorithm* OR classifier* OR computer-heuristics* OR cross-validation* OR data-mining* OR fuzzy-system* OR iterative-closest-point* OR k-nearest-neighbor* OR kernel-method* OR knowledge-discovery* OR memristor* OR molecular-docking* OR multicriteria-decision-analysis* OR multifactor-dimensionality-reduction* OR online-analytical-processing* OR outlier-detection* OR perceptron* OR radial-basis-function* OR random-forest* OR recursive-feature-elimination* OR recursive-partitioning* OR rough-set* OR convolution*-neur*-network* OR ((ambient OR artificial*) NEAR/2 intelligen*) OR (automat* NEAR/2 reasoning*) OR (Computer NEAR/2 heuristic*) OR Multicriteria-decision-analysis OR ((predict* OR forecast* OR prognos*) NEAR/2 (model* OR automat* OR algorit*)))) |
