# Supplementary material for: Using Artificial Intelligence to Predict Intracranial Hypertension in Patients After Traumatic Brain Injury: A Systematic Review
Source: Neurocrit Care. 2024 Jan 11;41(1):285–96. doi: 10.1007/s12028-023-01910-2 (PMC11335950; doi:10.1007/s12028-023-01910-2)
Supplement: Supplementary file 2 — Supplementary file2 (DOCX 96 kb) [file 12028_2023_1910_MOESM2_ESM.docx]

Supplementary file 2: PROBAST assessments

| **Study: Klauber MR et al. A model for predicting delayed intracranial hypertension following severe head injury. J Neurosurg. 1984 Oct; 61(4): 695-9** | | | |
| --- | --- | --- | --- |
| **Domain 1: Participants** | | | |
| **A. Risk of Bias** | | | |
| *Describe the sources of data and criteria for participant selection:*    “*In an effort to study severe head injury in the United States, the National Institute of Neurological and Communicative Disorders and Stroke and six medical centers joined in a cooperative prospective study. In the pilot phase of this prospective study (January 1, 1980, through May 31, 1982), data were collected on 581 patients. Entry into the Data Bank is restricted to trauma patients of all ages who either had a Glasgow Coma*  *Scale (GCS) score of 8 or less following resuscitation at a Center hospital or deteriorated to that level within 48 hours of injury.*” | | | |
|  | | Dev | Val |
| 1.1 Were the appropriate data sources used, e.g. cohort, RCT or nested case-control study data? | | Probably Yes | Probably Yes |
| 1.2 Were all inclusions and exclusions of participants appropriate? | | Probably Yes | Probably Yes |
| **Risk of bias introduced by selection of participants:** | **Risk:**  (low/high/unclear) | Low | Low |
| *Rationale of bias rating:*  Selection process explained. | | | |
| **B. Applicability** | | | |
| *Describe included participants, setting and dates:*  See A. | | | |
| **Concern that the included participants and setting do not match the review question** | **Concern:**  (low/high/unclear) | Low | Low |
| *Rationale of applicability rating:*  Participants are TBI patients. | | | |
| **Domain 2: Predictors** |  |  |  |
| **A. Risk of Bias** |  |  |  |
| *List and describe predictors included in the final model, e.g., definition and timing of assessment:*  The presence/value of ICP peak value, abnormal ventricle size on CT, and hypotension, during the first 24 hours of admission. | | | |
|  | | Dev | Val |
| 2.1 Were predictors defined and assessed in a similar way for all participants? | | Yes | Yes |
| 2.2 Were predictor assessments made without knowledge of outcome data? | | Yes | Yes |
| 2.3 Are all predictors available at the time the model is intended to be used? | | Probably Yes | Probably Yes |
| **Risk of bias introduced by predictors or their assessment** | **Risk:**  (low/high/unclear) | Low | Low |
| *Rationale of bias rating:*  Used predictors are a reasonable choice. | | | |
| **B. Applicability** |  |  |  |
| **Concern that the definition, assessment, or timing of predictors in the model do not match the review questions** | **Concern:**  (low/high/unclear) | Low | Low |
| *Rationale of applicability rating:*  Used predictors are a reasonable choice. | | | |
| **Domain 3: Outcome** | | | |
| **A. Risk of Bias** | | | |
|  | | Dev | Val |
| 3.1 Was the outcome determined appropriately? | | No | No |
| 3.2 Was a pre-specified or standard outcome definition used? | | No | No |
| 3.3 Were predictors excluded from the outcome definition? | | Yes | Yes |
| 3.4 Was the outcome defined and determined in a similar way for all participants? | | Yes | Yes |
| 3.5 Was the outcome determined without knowledge of predictor information? | | Yes | Yes |
| 3.6 Was the time interval between predictor assessment and outcome determination appropriate? | | Probably Yes | Probably Yes |
| **Risk of bias introduced by the outcome or its determination** | **Risk:**  (low/high/unclear) | High | High |
| *Rationale of bias rating:*  Unclear definition of intracranial hypertension. | | | |
| **B. Applicability** | | | |
| *At what time point was the outcome determined:*  At the start of the study.  *If a composite outcome was used, describe the relative frequency/distribution of each contributing outcome:*  Highest ICP peak value during first 24 hours, in combination with (abnormal or normal) ventricle size and/or blood pressure values above or under 90 mmHg. | | | |
| **Concern that the outcome, its definition, timing or determination do not match the review question** | **Concern:**  (low/high/unclear) | High | High |
| Rationale of applicability rating:  Outcome is somewhat similar to what the review investigates, but no clear definition is used. | | | |
| **Domain 4: Analysis** | | | |
| **Risk of Bias** | | | |
| *Describe numbers of participants, number of candidate predictors, outcome events and events per candidate predictor:*  Training set: 156 patients. 55 events  Validation set: 93 patients. 29 events. | | | |
| *Describe how the model was developed (for example in regards to modelling technique (e.g. survival or logistic modelling), predictor selection, and risk group definition):*  Logistic regression. | | | |
| *Describe whether and how the model was validated, either internally (e.g., bootstrapping, cross validation, random split sample) or externally (e.g., temporal validation, geographical validation, different setting, different type of participants):*  This study internally validates the model with different patients from the same data bank.  “*This splitting of the sample into the two groups was made at a natural break point in data entry, which occurred at a time when the data collection instruments were revised.*” | | | |
| *Describe the performance measures of the model, e.g., (re)calibration, discrimination, (re)classification, net benefit, and whether they were adjusted for optimism:*  Accuracy, sensitivity, specificity. | | | |
| *Describe any participants who were excluded from the analysis:*  “*If information on any one of these variables was not present during the first 24 hours of a patient's admission, that patient was excluded from the analysis of that variable. That is, no attempt was made to estimate missing data based only on the information that was present.*” | | | |
| *Describe missing data on predictors and outcomes as well as methods used for missing data:*  See above. | | | |
|  | | Dev | Val |
| 4.1 Were there a reasonable number of participants with the outcome? | | Yes | Yes |
| 4.2 Were continuous and categorical predictors handled appropriately? | | Probably Yes | Probably Yes |
| 4.3 Were all enrolled participants included in the analysis? | | No | No |
| 4.4 Were participants with missing data handled appropriately? | | No | No |
| 4.5 Was selection of predictors based on univariable analysis avoided? | | No |  |
| 4.6 Were complexities in the data (e.g., censoring, competing risks, sampling of controls) accounted for appropriately? | | Probably Yes | Probably Yes |
| 4.7 Were relevant model performance measures evaluated appropriately? | | Yes | Yes |
| 4.8 Were model overfitting and optimism in model performance accounted for? | | No |  |
| 4.9 Do predictors and their assigned weights in the final model correspond to the results from multivariable analysis? | | No |  |
| **Risk of bias introduced by the analysis** | **Risk:**  (low/high/unclear) | High | High |
| *Rationale of bias rating:*  Patients with missing data were excluded. Validation data comprised of patients after instrument calibration, which may influence the collected predictor values. | | | |
| **Overall judgement about risk of bias and applicability of the prediction model evaluation** | | | |
| **Overall judgement of risk of bias** | **Risk:**  (low/high/unclear) | High | |
| *Summary of sources of potential bias:*  Domain 3 and 4. | | | |
| **Overall judgement of applicability** | **Concern:**  (low/high/unclear) | High | |
| *Summary of applicability concerns:*  This research matches the review question, although the outcome could have been specified better. | | | |

| **Study: Feng M et al. Utilization of temporal information for intracranial pressure development trend forecasting in traumatic brain injury. Annu Int Conf IEEE Eng Med Biol Soc. 2012; 2012: 3930-4** | | | |
| --- | --- | --- | --- |
| **Domain 1: Participants** | | | |
| **A. Risk of Bias** | | | |
| *Describe the sources of data and criteria for participant selection:*    “*This analytical study was conducted based on the monitoring data of TBI patients, who were admitted to the neurocritical care unit of a tertiary hospital between January 2002 to December 2007. In particular, 82 patients, who underwent invasive monitoring of ICP, MAP and PbtO2 for more than 24 consecutive hours and were connected to a*  *bedside computerized system, were selected for the study.”* | | | |
|  | | Dev | Val |
| 1.1 Were the appropriate data sources used, e.g. cohort, RCT or nested case-control study data? | | Probably Yes | Probably Yes |
| 1.2 Were all inclusions and exclusions of participants appropriate? | | Probably Yes | No |
| **Risk of bias introduced by selection of participants:** | **Risk:**  (low/high/unclear) | Low | High |
| *Rationale of bias rating:*  Patient selection process described, although it is not clear if *every* eligible patient from the specified timeframe was included. | | | |
| **B. Applicability** | | | |
| *Describe included participants, setting and dates:*  “*82 traumatic brain injuries patients, who were admitted between 2002 to 2007 and were continuously monitored on ICP for more than 24 hours, are selected for the study.”* | | | |
| **Concern that the included participants and setting do not match the review question** | **Concern:**  (low/high/unclear) | Low | Low |
| *Rationale of applicability rating:*  Participants are TBI patients. | | | |
| **Domain 2: Predictors** |  |  |  |
| **A. Risk of Bias** |  |  |  |
| *List and describe predictors included in the final model, e.g., definition and timing of assessment:*  Intracranial pressure, mean arterial pressure, brain tissue oxygenation, pressure reactivity index.  “*ICP was contiously monitored using a fiber-optic intraparenchymal gauge (Codman and*  *Shurtleff, Taynham, MA), and Licox polarographic Clark-type microcatheters (Integra Neuroscience, Plainsboro, NJ) were inserted into peri-lesional brain tissues to measure the*  *brain temperature and PbtO2. MAP was measured through an arterial line from the radial artery using a standard pressure monitoring kit (Biosensors International Pte. Ltd., The Netherlands). The continuously monitored physiological readings were sampled and recorded every 5 sec via a computerized system. The PRx was calculated as a moving*  *correlation between the last 30 consecutive samples of ICP and MAP readings.”* | | | |
|  | | Dev | Val |
| 2.1 Were predictors defined and assessed in a similar way for all participants? | | Yes | Yes |
| 2.2 Were predictor assessments made without knowledge of outcome data? | | Yes | Yes |
| 2.3 Are all predictors available at the time the model is intended to be used? | | Probably Yes | Probably Yes |
| **Risk of bias introduced by predictors or their assessment** | **Risk:**  (low/high/unclear) | Low | Low |
| *Rationale of bias rating:*  Predictors are continuously measured or can easily be calculated. | | | |
| **B. Applicability** |  |  |  |
| **Concern that the definition, assessment, or timing of predictors in the model do not match the review questions** | **Concern:**  (low/high/unclear) | Low | Low |
| *Rationale of applicability rating:*  Used predictors are a reasonable choice. | | | |
| **Domain 3: Outcome** | | | |
| **A. Risk of Bias** | | | |
|  | | Dev | Val |
| 3.1 Was the outcome determined appropriately? | | Yes | Yes |
| 3.2 Was a pre-specified or standard outcome definition used? | | Yes | Yes |
| 3.3 Were predictors excluded from the outcome definition? | | No | No |
| 3.4 Was the outcome defined and determined in a similar way for all participants? | | Yes | Yes |
| 3.5 Was the outcome determined without knowledge of predictor information? | | Yes | Yes |
| 3.6 Was the time interval between predictor assessment and outcome determination appropriate? | | Probably Yes | Probably Yes |
| **Risk of bias introduced by the outcome or its determination** | **Risk:**  (low/high/unclear) | Low | Low |
| *Rationale of bias rating:*  The ICP is being used to predict the course of the ICP itself, but we do not think that this poses a risk of bias. | | | |
| **B. Applicability** | | | |
| *At what time point was the outcome determined:*  At the start of the study.  *If a composite outcome was used, describe the relative frequency/distribution of each contributing outcome:*  N/A. | | | |
| **Concern that the outcome, its definition, timing or determination do not match the review question** | **Concern:**  (low/high/unclear) | High | High |
| Rationale of applicability rating:  This study predicts whether the ICP will elevate, stay or reduce. This is not exactly the same as forecasting if the ICP will exceed a certain threshold, which is the actual review question. | | | |
| **Domain 4: Analysis** | | | |
| **Risk of Bias** | | | |
| *Describe numbers of participants, number of candidate predictors, outcome events and events per candidate predictor:*  82 patients were included. | | | |
| *Describe how the model was developed (for example in regards to modelling technique (e.g. survival or logistic modelling), predictor selection, and risk group definition):*  Aggregating One-Dependence Estimators (AODE), Ada-Bossting with Decision Tree (AdaBoost-J48), Bayesian Network with K2 & TAN (BayesNet-K2/TAN), Lazy Bayesian Rules (LBR), Logistic Regression (LogReg), Naïve Bayesian Classifier (Naive Bayes) and Support Vector Machine (SVM). | | | |
| *Describe whether and how the model was validated, either internally (e.g., bootstrapping, cross validation, random split sample) or externally (e.g., temporal validation, geographical validation, different setting, different type of participants):*  “*The forecasting performance was determined by a 10-fold cross-validation.”* | | | |
| *Describe the performance measures of the model, e.g., (re)calibration, discrimination, (re)classification, net benefit, and whether they were adjusted for optimism:*  Area Under the Receiver Operator Curve, F-measure. | | | |
| *Describe any participants who were excluded from the analysis:*  “*Note that necessary medical intervention were administered to the patients to maintain their ICP to be below 20 mmHg. These interventions usually induce a sudden reduction in ICP and thus interrupts our trend study. Therefore, to minimize the effect of intervention, only data points in between two intervention were used for the study.*” | | | |
| *Describe missing data on predictors and outcomes as well as methods used for missing data:*  Not mentioned. | | | |
|  | | Dev | Val |
| 4.1 Were there a reasonable number of participants with the outcome? | | No | No |
| 4.2 Were continuous and categorical predictors handled appropriately? | | Probably Yes | Probably Yes |
| 4.3 Were all enrolled participants included in the analysis? | | Yes | Yes |
| 4.4 Were participants with missing data handled appropriately? | | Yes | Yes |
| 4.5 Was selection of predictors based on univariable analysis avoided? | | Yes |  |
| 4.6 Were complexities in the data (e.g., censoring, competing risks, sampling of controls) accounted for appropriately? | | No | No |
| 4.7 Were relevant model performance measures evaluated appropriately? | | Yes | Yes |
| 4.8 Were model overfitting and optimism in model performance accounted for? | | No |  |
| 4.9 Do predictors and their assigned weights in the final model correspond to the results from multivariable analysis? | | Not described |  |
| **Risk of bias introduced by the analysis** | **Risk:**  (low/high/unclear) | N/A | High |
| *Rationale of bias rating:*  Relatively small number of participants. Unclear how many samples per patient were taken. No investigation of possible overfitting. | | | |
| **Overall judgement about risk of bias and applicability of the prediction model evaluation** | | | |
| **Overall judgement of risk of bias** | **Risk:**  (low/high/unclear) | High | |
| *Summary of sources of potential bias:*  Domain 1, 3, and 4. | | | |
| **Overall judgement of applicability** | **Concern:**  (low/high/unclear) | High | |
| *Summary of applicability concerns:*  The investigated outcome is relevant for our review, but is not exactly the same. | | | |

| **Study: Güiza F et al. Novel methods to predict increased intracranial pressure during intensive care and long-term neurologic outcome after traumatic brain injury: development and validation in a multicenter dataset. Crit Care Med. 2013 Feb; 41(2): 554-64** | | | |
| --- | --- | --- | --- |
| **Domain 1: Participants** | | | |
| **A. Risk of Bias** | | | |
| *Describe the sources of data and criteria for participant selection:*  “*The Brain-IT (17) database contains validated information of*  *264 TBI patients admitted to 22 neuro-ICUs in 11 European*  *countries between March 2003 and July 2005.*”  “*Complete data records were initially only available for 178 patients and later extended with data for the remaining 61 patients. The first set of patients was used as development cohort and the remaining as validation cohort.*” | | | |
|  | | Dev | Val |
| 1.1 Were the appropriate data sources used, e.g. cohort, RCT or nested case-control study data? | | Yes | Yes |
| 1.2 Were all inclusions and exclusions of participants appropriate? | | Probably Yes | No |
| **Risk of bias introduced by selection of participants:** | **Risk:**  (low/high/unclear) | Low | High |
| *Rationale of bias rating:*  Patient selection explained, but unclear extension of patient data. | | | |
| **B. Applicability** | | | |
| *Describe included participants, setting and dates:*  Development cohort: Median (IQR) age: 33,1 (19-49)  Percentage of male participants: 80,9  Increased ICP episodes per patient, median (IQR): 1 (0-6)  Validation cohort:  Median (IQR) age: 24 (13-44)  Percentage of male participants: 77,1  Increased ICP episodes per patient, median (IQR): 1 (0-6) | | | |
| **Concern that the included participants and setting do not match the review question** | **Concern:**  (low/high/unclear) | Low | Low |
| *Rationale of applicability rating:*  Participants are TBI patients. | | | |
| **Domain 2: Predictors** |  |  |  |
| **A. Risk of Bias** |  |  |  |
| *List and describe predictors included in the final model, e.g., definition and timing of assessment:*  “*We developed a model to predict increased intracranial pressure episodes 30 mins in advance, by using the dynamic characteristics of continuous intracranial pressure and mean arterial pressure monitoring.*” | | | |
|  | | Dev | Val |
| 2.1 Were predictors defined and assessed in a similar way for all participants? | | Yes | Yes |
| 2.2 Were predictor assessments made without knowledge of outcome data? | | Yes | Yes |
| 2.3 Are all predictors available at the time the model is intended to be used? | | Yes | Yes |
| **Risk of bias introduced by predictors or their assessment** | **Risk:**  (low/high/unclear) | Low | Low |
| *Rationale of bias rating:*  Reasonable choice for predictors, which are frequently assessed. | | | |
| **B. Applicability** |  |  |  |
| **Concern that the definition, assessment, or timing of predictors in the model do not match the review questions** | **Concern:**  (low/high/unclear) | Low | Low |
| *Rationale of applicability rating:*  Used predictors are a reasonable choice. | | | |
| **Domain 3: Outcome** | | | |
| **A. Risk of Bias** | | | |
|  | | Dev | Val |
| 3.1 Was the outcome determined appropriately? | | Yes | Yes |
| 3.2 Was a pre-specified or standard outcome definition used? | | Yes | Yes |
| 3.3 Were predictors excluded from the outcome definition? | | No | No |
| 3.4 Was the outcome defined and determined in a similar way for all participants? | | Yes | Yes |
| 3.5 Was the outcome determined without knowledge of predictor information? | | Yes | Yes |
| 3.6 Was the time interval between predictor assessment and outcome determination appropriate? | | Probably Yes | Probably Yes |
| **Risk of bias introduced by the outcome or its determination** | **Risk:**  (low/high/unclear) | Low | Low |
| *Rationale of bias rating:*  The ICP is being used to predict the course of the ICP itself, but we do not think that this poses a risk of bias. | | | |
| **B. Applicability** | | | |
| *At what time point was the outcome determined:*  At the start of the study.  *If a composite outcome was used, describe the relative frequency/distribution of each contributing outcome:*  N/A. | | | |
| **Concern that the outcome, its definition, timing or determination do not match the review question** | **Concern:**  (low/high/unclear) | Low | Low |
| Rationale of applicability rating:  Outcome is exactly what this review aims to investigate. | | | |
| **Domain 4: Analysis** | | | |
| **Risk of Bias** | | | |
| *Describe numbers of participants, number of candidate predictors, outcome events and events per candidate predictor:*  178 patients in the development cohort (106 (61%) patients with elevated ICP episodes, 982/2677 (37%) elevated ICP instances), 61 patients in the validation cohort (33 (54%) patients with elevated ICP episodes, 392/1135 (35%) elevated ICP instances). | | | |
| *Describe how the model was developed (for example in regards to modelling technique (e.g. survival or logistic modelling), predictor selection, and risk group definition):*  Gaussian Processes was used. | | | |
| *Describe whether and how the model was validated, either internally (e.g., bootstrapping, cross validation, random split sample) or externally (e.g., temporal validation, geographical validation, different setting, different type of participants):*  This study internally validates the model using a different set of patients, but from the same dataset. | | | |
| *Describe the performance measures of the model, e.g., (re)calibration, discrimination, (re)classification, net benefit, and whether they were adjusted for optimism:*  Area Under the Receiver Operator Curve, accuracy, sensitivity, specificity. | | | |
| *Describe any participants who were excluded from the analysis:*  Patients without complete data records. | | | |
| *Describe missing data on predictors and outcomes as well as methods used for missing data:*  Not mentioned. | | | |
|  | | Dev | Val |
| 4.1 Were there a reasonable number of participants with the outcome? | | Yes | Yes |
| 4.2 Were continuous and categorical predictors handled appropriately? | | Probably Yes | Probably Yes |
| 4.3 Were all enrolled participants included in the analysis? | | Yes | Yes |
| 4.4 Were participants with missing data handled appropriately? | | Probably Yes | Probably Yes |
| 4.5 Was selection of predictors based on univariable analysis avoided? | | Probably Yes |  |
| 4.6 Were complexities in the data (e.g., censoring, competing risks, sampling of controls) accounted for appropriately? | | Probably Yes | Probably Yes |
| 4.7 Were relevant model performance measures evaluated appropriately? | | Yes | Yes |
| 4.8 Were model overfitting and optimism in model performance accounted for? | | Yes |  |
| 4.9 Do predictors and their assigned weights in the final model correspond to the results from multivariable analysis? | | Not described |  |
| **Risk of bias introduced by the analysis** | **Risk:**  (low/high/unclear) | Low | Low |
| *Rationale of bias rating:*  The only thing missing would be stating how many samples were taken per patient. | | | |
| **Overall judgement about risk of bias and applicability of the prediction model evaluation** | | | |
| **Overall judgement of risk of bias** | **Risk:**  (low/high/unclear) | Low | |
| *Summary of sources of potential bias:*  No domains. | | | |
| **Overall judgement of applicability** | **Concern:**  (low/high/unclear) | Low | |
| *Summary of applicability concerns:*  Research matches the review question. | | | |

| **Study: Beckers M et al. External validation of an early warning alert for elevated intracranial pressure in the Avert-IT database. Crit Care. 2014; 18: S163** | | | |
| --- | --- | --- | --- |
| **Domain 1: Participants** | | | |
| **A. Risk of Bias** | | | |
| *Describe the sources of data and criteria for participant selection:*    Avert-IT database; a cohort of adult TBI patients. “*A retrospective analysis of physiological data collected at the minute resolution, from 43 adult patients from the AVERT-IT project. A total of 67 episodes of ICP above 30 mmHg lasting at least 10 minutes were identified in this cohort.*” | | | |
|  | | Dev | Val |
| 1.1 Were the appropriate data sources used, e.g. cohort, RCT or nested case-control study data? | | N/A | Probably Yes |
| 1.2 Were all inclusions and exclusions of participants appropriate? | | N/A | No |
| **Risk of bias introduced by selection of participants:** | **Risk:**  (low/high/unclear) | N/A | High |
| *Rationale of bias rating:*  Unclear patient selection process. | | | |
| **B. Applicability** | | | |
| *Describe included participants, setting and dates:*  “*Here we present external validation results of this model, on a more*  *recent cohort of adult TBI patients from the AVERT-IT project.*” | | | |
| **Concern that the included participants and setting do not match the review question** | **Concern:**  (low/high/unclear) | N/A | Low |
| *Rationale of applicability rating:*  Participants are TBI patients. | | | |
| **Domain 2: Predictors** |  |  |  |
| **A. Risk of Bias** |  |  |  |
| *List and describe predictors included in the final model, e.g., definition and timing of assessment:*  “*Previously we developed a model to predict increased ICP, 30 minutes*  *in advance, using the dynamic characteristics of routinely monitored*  *minute-by-minute ICP and mean arterial blood pressure (MAP) signals.*” | | | |
|  | | Dev | Val |
| 2.1 Were predictors defined and assessed in a similar way for all participants? | | N/A | Yes |
| 2.2 Were predictor assessments made without knowledge of outcome data? | | N/A | Yes |
| 2.3 Are all predictors available at the time the model is intended to be used? | | N/A | Yes |
| **Risk of bias introduced by predictors or their assessment** | **Risk:**  (low/high/unclear) | N/A | Low |
| *Rationale of bias rating:*  Predictors were determined in a previous study. | | | |
| **B. Applicability** |  |  |  |
| **Concern that the definition, assessment, or timing of predictors in the model do not match the review questions** | **Concern:**  (low/high/unclear) | N/A | Low |
| *Rationale of applicability rating:*  Used predictors are a reasonable choice. | | | |
| **Domain 3: Outcome** | | | |
| **A. Risk of Bias** | | | |
|  | | Dev | Val |
| 3.1 Was the outcome determined appropriately? | | N/A | Yes |
| 3.2 Was a pre-specified or standard outcome definition used? | | N/A | Yes |
| 3.3 Were predictors excluded from the outcome definition? | | N/A | Yes |
| 3.4 Was the outcome defined and determined in a similar way for all participants? | | N/A | Yes |
| 3.5 Was the outcome determined without knowledge of predictor information? | | N/A | Yes |
| 3.6 Was the time interval between predictor assessment and outcome determination appropriate? | | N/A | Probably Yes |
| **Risk of bias introduced by the outcome or its determination** | **Risk:**  (low/high/unclear) | N/A | Low |
| *Rationale of bias rating:*  Clear outcome definition was used. | | | |
| **B. Applicability** | | | |
| *At what time point was the outcome determined:*  In a previous study.  *If a composite outcome was used, describe the relative frequency/distribution of each contributing outcome:*  N/A. | | | |
| **Concern that the outcome, its definition, timing or determination do not match the review question** | **Concern:**  (low/high/unclear) | N/A | Low |
| Rationale of applicability rating:  Outcome is exactly what this review aims to investigate. | | | |
| **Domain 4: Analysis** | | | |
| **Risk of Bias** | | | |
| *Describe numbers of participants, number of candidate predictors, outcome events and events per candidate predictor:*  43 patients were included. “*A total of 67 episodes of ICP above 30 mmHg lasting at least 10 minutes were identified in this cohort.*” | | | |
| *Describe how the model was developed (for example in regards to modelling technique (e.g. survival or logistic modelling), predictor selection, and risk group definition):*  Gaussian Processes was used. “*Four-hour time series of ICP and MAP anteceding each episode by 30 minutes were analyzed. Additional time series not preceding elevated ICP episodes were used for validation.*” | | | |
| *Describe whether and how the model was validated, either internally (e.g., bootstrapping, cross validation, random split sample) or externally (e.g., temporal validation, geographical validation, different setting, different type of participants):*  This study externally validates a model developed in a previous study, using a different dataset. | | | |
| *Describe the performance measures of the model, e.g., (re)calibration, discrimination, (re)classification, net benefit, and whether they were adjusted for optimism:*  Area Under the Receiver Operator Curve. | | | |
| *Describe any participants who were excluded from the analysis:*  Not mentioned. | | | |
| *Describe missing data on predictors and outcomes as well as methods used for missing data:*  Not mentioned. | | | |
|  | | Dev | Val |
| 4.1 Were there a reasonable number of participants with the outcome? | | N/A | No |
| 4.2 Were continuous and categorical predictors handled appropriately? | | N/A | Probably Yes |
| 4.3 Were all enrolled participants included in the analysis? | | N/A | Yes |
| 4.4 Were participants with missing data handled appropriately? | | N/A | Probably Yes |
| 4.5 Was selection of predictors based on univariable analysis avoided? | | N/A |  |
| 4.6 Were complexities in the data (e.g., censoring, competing risks, sampling of controls) accounted for appropriately? | | N/A | No |
| 4.7 Were relevant model performance measures evaluated appropriately? | | N/A | Yes |
| 4.8 Were model overfitting and optimism in model performance accounted for? | | N/A |  |
| 4.9 Do predictors and their assigned weights in the final model correspond to the results from multivariable analysis? | | N/A |  |
| **Risk of bias introduced by the analysis** | **Risk:**  (low/high/unclear) | N/A | High |
| *Rationale of bias rating:*  Relatively small number of participants. Unclear how many samples per patient were taken, especially “control samples”. | | | |
| **Overall judgement about risk of bias and applicability of the prediction model evaluation** | | | |
| **Overall judgement of risk of bias** | **Risk:**  (low/high/unclear) | High | |
| *Summary of sources of potential bias:*  Domain 1 and 4. | | | |
| **Overall judgement of applicability** | **Concern:**  (low/high/unclear) | Low | |
| *Summary of applicability concerns:*  Although there is a high risk of bias, this research matches the review question. | | | |

| **Study: Myers RB et al. Predicting Intracranial Pressure and Brain Tissue Oxygen Crises in Patients With Severe Traumatic Brain Injury. Crit Care Med. 2016 Sep; 44(9): 1754-61** | | | |
| --- | --- | --- | --- |
| **Domain 1: Participants** | | | |
| **A. Risk of Bias** | | | |
| *Describe the sources of data and criteria for participant selection:*    “*We constructed a database of 874 subjects who experienced severe TBI. Patient data were collected between 1989–2000 and 2006–2013. All subjects were admitted to the*  *neurosurgical ICU of Ben Taub General Hospital located in Houston, TX.*” | | | |
|  | | Dev | Val |
| 1.1 Were the appropriate data sources used, e.g. cohort, RCT or nested case-control study data? | | Probably Yes | Probably Yes |
| 1.2 Were all inclusions and exclusions of participants appropriate? | | Probably Yes | No |
| **Risk of bias introduced by selection of participants:** | **Risk:**  (low/high/unclear) | Low | High |
| *Rationale of bias rating:*  Unclear patient selection process (gap between 2000 and 2006). | | | |
| **B. Applicability** | | | |
| *Describe included participants, setting and dates:*  “*Here we present external validation results of this model, on a more*  *recent cohort of adult TBI patients from the AVERT-IT project* *Data collected from subjects between 1989 and 1996 formed the study cohort; data between 1996 and 2000 comprised the model selection cohort; whereas data between 2006 and 2013 defined the validation cohort.*”  “*Because data were collected over a period of 24 years, changes in patient care practices*  *could impact model applicability. Our groups are well matched in terms of age, gender, and admission Glasgow Coma Scale. No attempt was made to create strictly matched groups because our primary aim was event prediction, not outcome comparisons between cohorts. Differences in mortality rates may be due to trauma causes and advancement of care practices. Models were constructed and tuned using the oldest of the recorded data and evaluated on temporally distinct and more recent data. If such models work well across this wide time range, it provides strong evidence that the models are robust to changes in care management, which may make them widely applicable.*” | | | |
| **Concern that the included participants and setting do not match the review question** | **Concern:**  (low/high/unclear) | Low | Low |
| *Rationale of applicability rating:*  See description. | | | |
| **Domain 2: Predictors** |  |  |  |
| **A. Risk of Bias** |  |  |  |
| *List and describe predictors included in the final model, e.g., definition and timing of assessment:*  Intracranial pressure, time since last intracranial hypertension event. | | | |
|  | | Dev | Val |
| 2.1 Were predictors defined and assessed in a similar way for all participants? | | Yes | Yes |
| 2.2 Were predictor assessments made without knowledge of outcome data? | | Yes | Yes |
| 2.3 Are all predictors available at the time the model is intended to be used? | | Yes | Yes |
| **Risk of bias introduced by predictors or their assessment** | **Risk:**  (low/high/unclear) | Low | Low |
| *Rationale of bias rating:*  Used predictors are a reasonable choice. | | | |
| **B. Applicability** |  |  |  |
| **Concern that the definition, assessment, or timing of predictors in the model do not match the review questions** | **Concern:**  (low/high/unclear) | Low | Low |
| *Rationale of applicability rating:*  Used predictors are a reasonable choice. | | | |
| **Domain 3: Outcome** | | | |
| **A. Risk of Bias** | | | |
|  | | Dev | Val |
| 3.1 Was the outcome determined appropriately? | | Yes | Yes |
| 3.2 Was a pre-specified or standard outcome definition used? | | Yes | Yes |
| 3.3 Were predictors excluded from the outcome definition? | | No | No |
| 3.4 Was the outcome defined and determined in a similar way for all participants? | | Yes | Yes |
| 3.5 Was the outcome determined without knowledge of predictor information? | | Yes | Yes |
| 3.6 Was the time interval between predictor assessment and outcome determination appropriate? | | Probably Yes | Probably Yes |
| **Risk of bias introduced by the outcome or its determination** | **Risk:**  (low/high/unclear) | Low | Low |
| *Rationale of bias rating:*  Clear outcome definition was used. | | | |
| **B. Applicability** | | | |
| *At what time point was the outcome determined:*  At the start of the study.  *If a composite outcome was used, describe the relative frequency/distribution of each contributing outcome:*  N/A. | | | |
| **Concern that the outcome, its definition, timing or determination do not match the review question** | **Concern:**  (low/high/unclear) | Low | Low |
| Rationale of applicability rating:  Outcome is exactly what this review aims to investigate. | | | |
| **Domain 4: Analysis** | | | |
| **Risk of Bias** | | | |
| *Describe numbers of participants, number of candidate predictors, outcome events and events per candidate predictor:*  Training cohort: 368 patients (43353 samples, 5979 events (14%))  Validation cohort: 261 patients (38349 samples, 4025 events (10%)) | | | |
| *Describe how the model was developed (for example in regards to modelling technique (e.g. survival or logistic modelling), predictor selection, and risk group definition):*  The final model was Autoregressive Ordinal-Regression (AR-OR). | | | |
| *Describe whether and how the model was validated, either internally (e.g., bootstrapping, cross validation, random split sample) or externally (e.g., temporal validation, geographical validation, different setting, different type of participants):*  This study internally validates the model using data collected in a later time period. 10-fold cross validation was used. | | | |
| *Describe the performance measures of the model, e.g., (re)calibration, discrimination, (re)classification, net benefit, and whether they were adjusted for optimism:*  Area Under the Receiver Operator Curve. | | | |
| *Describe any participants who were excluded from the analysis:*  Not mentioned. | | | |
| *Describe missing data on predictors and outcomes as well as methods used for missing data:*  Excluded physiologically impossible values, interpolated missing data and used a smoothing filter. | | | |
|  | | Dev | Val |
| 4.1 Were there a reasonable number of participants with the outcome? | | No | No |
| 4.2 Were continuous and categorical predictors handled appropriately? | | Probably Yes | Probably Yes |
| 4.3 Were all enrolled participants included in the analysis? | | Yes | Yes |
| 4.4 Were participants with missing data handled appropriately? | | Yes | Yes |
| 4.5 Was selection of predictors based on univariable analysis avoided? | | Unclear |  |
| 4.6 Were complexities in the data (e.g., censoring, competing risks, sampling of controls) accounted for appropriately? | | Probably Yes | Probably Yes |
| 4.7 Were relevant model performance measures evaluated appropriately? | | Yes | Yes |
| 4.8 Were model overfitting and optimism in model performance accounted for? | | No |  |
| 4.9 Do predictors and their assigned weights in the final model correspond to the results from multivariable analysis? | | Probably No |  |
| **Risk of bias introduced by the analysis** | **Risk:**  (low/high/unclear) | High | High |
| *Rationale of bias rating:*  Relatively small percentage of samples with outcome. Unclear how many samples per patient were taken. | | | |
| **Overall judgement about risk of bias and applicability of the prediction model evaluation** | | | |
| **Overall judgement of risk of bias** | **Risk:**  (low/high/unclear) | High | |
| *Summary of sources of potential bias:*  Domain 4. | | | |
| **Overall judgement of applicability** | **Concern:**  (low/high/unclear) | Low | |
| *Summary of applicability concerns:*  Although there is a high risk of bias, this research matches the review question. | | | |

| **Study: Güiza F et al. Early Detection of Increased Intracranial Pressure Episodes in Traumatic Brain Injury: External Validation in an Adult and in a Pediatric Cohort. Crit Care Med. 2017 Mar; 45(3): e316-e320** | | | |
| --- | --- | --- | --- |
| **Domain 1: Participants** | | | |
| **A. Risk of Bias** | | | |
| *Describe the sources of data and criteria for participant selection:*    “*The adult validation cohort comprised recent traumatic brain injury patients from San Gerardo Hospital in Monza (n = 50), Leuven University Hospital (n = 26), Antwerp*  *University Hospital (n = 19), Tübingen University Hospital (n = 18), and Southern General Hospital in Glasgow (n = 8). The pediatric validation cohort comprised patients from neurosurgical and intensive care centers in Edinburgh and Newcastle (n = 79).*” | | | |
|  | | Dev | Val |
| 1.1 Were the appropriate data sources used, e.g. cohort, RCT or nested case-control study data? | | N/A | Yes |
| 1.2 Were all inclusions and exclusions of participants appropriate? | | N/A | No |
| **Risk of bias introduced by selection of participants:** | **Risk:**  (low/high/unclear) | N/A | High |
| *Rationale of bias rating:*  Unclear patient selection process. | | | |
| **B. Applicability** | | | |
| *Describe included participants, setting and dates:*  Adult cohort:  Age, median (IQR): 50 (28,5-65)  % male participants: 78  Glasgow coma scale, median (IQR): 7 (3-12)  Pediatric cohort:  Age, median (IQR): 10,4 (7,5-14,2)  % male participants: 74  Glasgow coma scale, median (IQR): 6 (5-8) | | | |
| **Concern that the included participants and setting do not match the review question** | **Concern:**  (low/high/unclear) | N/A | Low |
| *Rationale of applicability rating:*  Participants are TBI patients. | | | |
| **Domain 2: Predictors** |  |  |  |
| **A. Risk of Bias** |  |  |  |
| *List and describe predictors included in the final model, e.g., definition and timing of assessment:*  Mean arterial pressure and intracranial pressure; the same as the predictors used in the previously developed model. | | | |
|  | | Dev | Val |
| 2.1 Were predictors defined and assessed in a similar way for all participants? | | N/A | Yes |
| 2.2 Were predictor assessments made without knowledge of outcome data? | | N/A | Yes |
| 2.3 Are all predictors available at the time the model is intended to be used? | | N/A | Yes |
| **Risk of bias introduced by predictors or their assessment** | **Risk:**  (low/high/unclear) | N/A | Low |
| *Rationale of bias rating:*  Predictors were determined in a previous study. | | | |
| **B. Applicability** |  |  |  |
| **Concern that the definition, assessment, or timing of predictors in the model do not match the review questions** | **Concern:**  (low/high/unclear) | N/A | Low |
| *Rationale of applicability rating:*  Used predictors are a reasonable choice. | | | |
| **Domain 3: Outcome** | | | |
| **A. Risk of Bias** | | | |
|  | | Dev | Val |
| 3.1 Was the outcome determined appropriately? | | N/A | Yes |
| 3.2 Was a pre-specified or standard outcome definition used? | | N/A | Yes |
| 3.3 Were predictors excluded from the outcome definition? | | N/A | Yes |
| 3.4 Was the outcome defined and determined in a similar way for all participants? | | N/A | Yes |
| 3.5 Was the outcome determined without knowledge of predictor information? | | N/A | Yes |
| 3.6 Was the time interval between predictor assessment and outcome determination appropriate? | | N/A | Probably Yes |
| **Risk of bias introduced by the outcome or its determination** | **Risk:**  (low/high/unclear) | N/A | Low |
| *Rationale of bias rating:*  Clear outcome definition was used. | | | |
| **B. Applicability** | | | |
| *At what time point was the outcome determined:*  In a previous study.  *If a composite outcome was used, describe the relative frequency/distribution of each contributing outcome:*  N/A. | | | |
| **Concern that the outcome, its definition, timing or determination do not match the review question** | **Concern:**  (low/high/unclear) | N/A | Low |
| Rationale of applicability rating:  Outcome is exactly what this review aims to investigate. | | | |
| **Domain 4: Analysis** | | | |
| **Risk of Bias** | | | |
| *Describe numbers of participants, number of candidate predictors, outcome events and events per candidate predictor:*  Adult cohort:  “*There were 231 instances of 4-hour time series of ICP and MAP leading to increased ICP episodes within the 30-minute time horizon, with at least one episode occurring in 41 patients; or equivalently an approximate average instance-to-patient ratio of 6:1. Additional 820 instances of 4-hour time series that did not precede increased ICP episodes within the 30-minute horizon were randomly selected from all available instances from the 121 patients, thus preserving the average instance-to-patient ratio.*”  Pediatric cohort:  “*There were 811 instances preceding increased ICP episodes, with at least one episode occurring in 49 patients. To preserve the average instance-to-patient ratio in this case, additional 1,408 instances not preceding hypertension episodes were randomly sampled from all 79 patients.*” | | | |
| *Describe how the model was developed (for example in regards to modelling technique (e.g. survival or logistic modelling), predictor selection, and risk group definition):*  Gaussian Processes was used; model was developed in a previous study. | | | |
| *Describe whether and how the model was validated, either internally (e.g., bootstrapping, cross validation, random split sample) or externally (e.g., temporal validation, geographical validation, different setting, different type of participants):*  This study externally validates a model developed in a previous study, using a new dataset of patients. | | | |
| *Describe the performance measures of the model, e.g., (re)calibration, discrimination, (re)classification, net benefit, and whether they were adjusted for optimism:*  Area Under the Receiver Operator Curve, accuracy, sensitivity, specificity. | | | |
| *Describe any participants who were excluded from the analysis:*  Patients without complete data records. | | | |
| *Describe missing data on predictors and outcomes as well as methods used for missing data:*  Not mentioned. | | | |
|  | | Dev | Val |
| 4.1 Were there a reasonable number of participants with the outcome? | | N/A | Yes |
| 4.2 Were continuous and categorical predictors handled appropriately? | | N/A | Probably Yes |
| 4.3 Were all enrolled participants included in the analysis? | | Yes | Yes |
| 4.4 Were participants with missing data handled appropriately? | | N/A | Probably Yes |
| 4.5 Was selection of predictors based on univariable analysis avoided? | | N/A |  |
| 4.6 Were complexities in the data (e.g., censoring, competing risks, sampling of controls) accounted for appropriately? | | N/A | Yes |
| 4.7 Were relevant model performance measures evaluated appropriately? | | N/A | Yes |
| 4.8 Were model overfitting and optimism in model performance accounted for? | | N/A |  |
| 4.9 Do predictors and their assigned weights in the final model correspond to the results from multivariable analysis? | | N/A |  |
| **Risk of bias introduced by the analysis** | **Risk:**  (low/high/unclear) | N/A | Low |
| *Rationale of bias rating:*  No clear statement regarding potential missing data. | | | |
| **Overall judgement about risk of bias and applicability of the prediction model evaluation** | | | |
| **Overall judgement of risk of bias** | **Risk:**  (low/high/unclear) | High | |
| *Summary of sources of potential bias:*  Domain 1. | | | |
| **Overall judgement of applicability** | **Concern:**  (low/high/unclear) | Low | |
| *Summary of applicability concerns:*  Although there is a high risk of bias, this research matches the review question. | | | |

| **Study: Carra G et al. Prediction model for intracranial hypertension demonstrates robust performance during external validation on the CENTER-TBI dataset. Intensive Care Med. 2020 Oct 1** | | | |
| --- | --- | --- | --- |
| **Domain 1: Participants** | | | |
| **A. Risk of Bias** | | | |
| *Describe the sources of data and criteria for participant selection:*    “*The validation dataset included 257 patients with TBI, who were recruited prospectively between 2015 and 2017 as part of the CENTER-TBI high-resolution ICU monitoring cohort*” | | | |
|  | | Dev | Val |
| 1.1 Were the appropriate data sources used, e.g. cohort, RCT or nested case-control study data? | | N/A | Probably Yes |
| 1.2 Were all inclusions and exclusions of participants appropriate? | | N/A | No |
| **Risk of bias introduced by selection of participants:** | **Risk:**  (low/high/unclear) | N/A | High |
| *Rationale of bias rating:*  Unclear patient selection process. | | | |
| **B. Applicability** | | | |
| *Describe included participants, setting and dates:*  “*Patients with TBI, who were recruited prospectively between 2015 and 2017 as part of the CENTER-TBI high-resolution ICU monitoring cohort.*”  Median (IQR) age: 47 (30-61),  Male participants (%): 208 (81)  Glasgow Coma Scale median (IQR): 6 (3-10) | | | |
| **Concern that the included participants and setting do not match the review question** | **Concern:**  (low/high/unclear) | N/A | Low |
| *Rationale of applicability rating:*  Participants are TBI patients. | | | |
| **Domain 2: Predictors** |  |  |  |
| **A. Risk of Bias** |  |  |  |
| *List and describe predictors included in the final model, e.g., definition and timing of assessment:*  The model only requires continuous ICP and mean arterial blood pressure signals. | | | |
|  | | Dev | Val |
| 2.1 Were predictors defined and assessed in a similar way for all participants? | | N/A | Yes |
| 2.2 Were predictor assessments made without knowledge of outcome data? | | N/A | Yes |
| 2.3 Are all predictors available at the time the model is intended to be used? | | N/A | Yes |
| **Risk of bias introduced by predictors or their assessment** | **Risk:**  (low/high/unclear) | N/A | Low |
| *Rationale of bias rating:*  Predictors were determined in a previous study. | | | |
| **B. Applicability** |  |  |  |
| **Concern that the definition, assessment, or timing of predictors in the model do not match the review questions** | **Concern:**  (low/high/unclear) | N/A | Low |
| *Rationale of applicability rating:*  Used predictors are a reasonable choice. | | | |
| **Domain 3: Outcome** | | | |
| **A. Risk of Bias** | | | |
|  | | Dev | Val |
| 3.1 Was the outcome determined appropriately? | | N/A | Yes |
| 3.2 Was a pre-specified or standard outcome definition used? | | N/A | Yes |
| 3.3 Were predictors excluded from the outcome definition? | | N/A | Yes |
| 3.4 Was the outcome defined and determined in a similar way for all participants? | | N/A | Yes |
| 3.5 Was the outcome determined without knowledge of predictor information? | | N/A | Yes |
| 3.6 Was the time interval between predictor assessment and outcome determination appropriate? | | N/A | Probably Yes |
| **Risk of bias introduced by the outcome or its determination** | **Risk:**  (low/high/unclear) | N/A | Low |
| *Rationale of bias rating:*  Clear outcome definition was used. | | | |
| **B. Applicability** | | | |
| *At what time point was the outcome determined:*  In a previous study.  *If a composite outcome was used, describe the relative frequency/distribution of each contributing outcome:*  N/A. | | | |
| **Concern that the outcome, its definition, timing or determination do not match the review question** | **Concern:**  (low/high/unclear) | N/A | Low |
| Rationale of applicability rating:  Outcome is exactly what this review aims to investigate. | | | |
| **Domain 4: Analysis** | | | |
| **Risk of Bias** | | | |
| *Describe numbers of participants, number of candidate predictors, outcome events and events per candidate predictor:*  257 patients were included. No amount of outcome events or events per candidate predictor were mentioned. | | | |
| *Describe how the model was developed (for example in regards to modelling technique (e.g. survival or logistic modelling), predictor selection, and risk group definition):*  Gaussian Processes was used. | | | |
| *Describe whether and how the model was validated, either internally (e.g., bootstrapping, cross validation, random split sample) or externally (e.g., temporal validation, geographical validation, different setting, different type of participants):*  This study externally validates a model developed in a previous study, using a different dataset. | | | |
| *Describe the performance measures of the model, e.g., (re)calibration, discrimination, (re)classification, net benefit, and whether they were adjusted for optimism:*  Area Under the Receiver Operator Curve, accuracy, sensitivity, specificity. | | | |
| *Describe any participants who were excluded from the analysis:*  Not mentioned. | | | |
| *Describe missing data on predictors and outcomes as well as methods used for missing data:*  Not mentioned. | | | |
|  | | Dev | Val |
| 4.1 Were there a reasonable number of participants with the outcome? | | N/A | Yes |
| 4.2 Were continuous and categorical predictors handled appropriately? | | N/A | Probably Yes |
| 4.3 Were all enrolled participants included in the analysis? | | N/A | Probably Yes |
| 4.4 Were participants with missing data handled appropriately? | | N/A | Probably Yes |
| 4.5 Was selection of predictors based on univariable analysis avoided? | | N/A |  |
| 4.6 Were complexities in the data (e.g., censoring, competing risks, sampling of controls) accounted for appropriately? | | N/A | No |
| 4.7 Were relevant model performance measures evaluated appropriately? | | N/A | Yes |
| 4.8 Were model overfitting and optimism in model performance accounted for? | | N/A |  |
| 4.9 Do predictors and their assigned weights in the final model correspond to the results from multivariable analysis? | | N/A |  |
| **Risk of bias introduced by the analysis** | **Risk:**  (low/high/unclear) | N/A | High |
| *Rationale of bias rating:*  Unclear how many samples per patient were taken and used in total. | | | |
| **Overall judgement about risk of bias and applicability of the prediction model evaluation** | | | |
| **Overall judgement of risk of bias** | **Risk:**  (low/high/unclear) | High | |
| *Summary of sources of potential bias:*  Domain 1 and 4. | | | |
| **Overall judgement of applicability** | **Concern:**  (low/high/unclear) | Low | |
| *Summary of applicability concerns:*  Although there is a high risk of bias, this research matches the review question. | | | |

| **Study: Wijayatunga P et al. Probabilistic prediction of increased intracranial pressure in patients with severe traumatic brain injury. Sci Rep. 2022 Jun 10; 12(1): 9600** | | | |
| --- | --- | --- | --- |
| **Domain 1: Participants** | | | |
| **A. Risk of Bias** | | | |
| *Describe the sources of data and criteria for participant selection:*    “*29 patients suffering from severe TBI and treated with neuro-intensive care at Umeå University Hospital. All patients were prospectively recruited from January 2015 to December 2017. Inclusion criteria were persons of all ages with a clinical diagnosis of severe TBI and an indication for CT that presented to the hospital within 24 h of injury.*  *Patients with severe preexisting neurological disorders that would confound outcome assessments were excluded.*” | | | |
|  | | Dev | Val |
| 1.1 Were the appropriate data sources used, e.g. cohort, RCT or nested case-control study data? | | Yes | Yes |
| 1.2 Were all inclusions and exclusions of participants appropriate? | | Yes | Yes |
| **Risk of bias introduced by selection of participants:** | **Risk:**  (low/high/unclear) | Low | Low |
| *Rationale of bias rating:*  Clear patient selection process. | | | |
| **B. Applicability** | | | |
| *Describe included participants, setting and dates:*  “*29 patients suffering from severe TBI and treated with neuro-intensive care at Umeå University Hospital. All patients were prospectively recruited from January 2015 to December 2017.*” | | | |
| **Concern that the included participants and setting do not match the review question** | **Concern:**  (low/high/unclear) | Low | Low |
| *Rationale of applicability rating:*  Participants are TBI patients. | | | |
| **Domain 2: Predictors** |  |  |  |
| **A. Risk of Bias** |  |  |  |
| *List and describe predictors included in the final model, e.g., definition and timing of assessment:*  “*The model is generalizable to any number of predictor variables but can be simplified when predictions are to be based on ICP data streams alone.*” | | | |
|  | | Dev | Val |
| 2.1 Were predictors defined and assessed in a similar way for all participants? | | Yes | Yes |
| 2.2 Were predictor assessments made without knowledge of outcome data? | | Yes | Yes |
| 2.3 Are all predictors available at the time the model is intended to be used? | | Yes | Yes |
| **Risk of bias introduced by predictors or their assessment** | **Risk:**  (low/high/unclear) | Low | Low |
| *Rationale of bias rating:*  ICP itself is used as predictor, which is not necessarily a problem. | | | |
| **B. Applicability** |  |  |  |
| **Concern that the definition, assessment, or timing of predictors in the model do not match the review questions** | **Concern:**  (low/high/unclear) | Low | Low |
| *Rationale of applicability rating:*  Used predictor is a reasonable choice. | | | |
| **Domain 3: Outcome** | | | |
| **A. Risk of Bias** | | | |
|  | | Dev | Val |
| 3.1 Was the outcome determined appropriately? | | No | No |
| 3.2 Was a pre-specified or standard outcome definition used? | | Yes | Yes |
| 3.3 Were predictors excluded from the outcome definition? | | Yes | Yes |
| 3.4 Was the outcome defined and determined in a similar way for all participants? | | Yes | Yes |
| 3.5 Was the outcome determined without knowledge of predictor information? | | Yes | Yes |
| 3.6 Was the time interval between predictor assessment and outcome determination appropriate? | | Yes | Yes |
| **Risk of bias introduced by the outcome or its determination** | **Risk:**  (low/high/unclear) | High | High |
| *Rationale of bias rating:*  An ICP threshold was used, but without specification of minimal duration. | | | |
| **B. Applicability** | | | |
| *At what time point was the outcome determined:*  In the beginning of the study.  *If a composite outcome was used, describe the relative frequency/distribution of each contributing outcome:*  N/A. | | | |
| **Concern that the outcome, its definition, timing or determination do not match the review question** | **Concern:**  (low/high/unclear) | Low | Low |
| Rationale of applicability rating:  Outcome is what this study aims to investigate, although an ICP duration specification is missing. | | | |
| **Domain 4: Analysis** | | | |
| **Risk of Bias** | | | |
| *Describe numbers of participants, number of candidate predictors, outcome events and events per candidate predictor:*  29 patients were included. “*In total, the model building and testing were based on 4018 h of ICP recordings. The limit for dangerously high ICP levels was set to 20 mmHg, i.e., ICP<20 mmHg was classified as “1” (normal) and ICP≥20 mmHg was classified as “2” (severe). Of the time series data, 1.7% were found to be<20 mmHg (i.e., “ICP=1”) and 18.3% were ≥20 mmHg (“ICP=2”)..*” | | | |
| *Describe how the model was developed (for example in regards to modelling technique (e.g. survival or logistic modelling), predictor selection, and risk group definition):*  Naïve Bayes was used. “*A probabilistic model for the prediction of future ICP levels of individual patients was developed. The model is generalizable to any number of predictor variables but can be simplified when predictions are to be based on ICP data streams alone. The model is a more general dynamic version of the so-called naïve Bayes mode.*” | | | |
| *Describe whether and how the model was validated, either internally (e.g., bootstrapping, cross validation, random split sample) or externally (e.g., temporal validation, geographical validation, different setting, different type of participants):*  This study internally validates the model using leave-one-out cross-validation. | | | |
| *Describe the performance measures of the model, e.g., (re)calibration, discrimination, (re)classification, net benefit, and whether they were adjusted for optimism:*  Accuracy, sensitivity, specificity. | | | |
| *Describe any participants who were excluded from the analysis:*  Not mentioned. | | | |
| *Describe missing data on predictors and outcomes as well as methods used for missing data:*  “*A software developed in Matlab was applied to identify and remove all artifacts lasting longer than three seconds (in total, 2.5% of the data were removed).*” | | | |
|  | | Dev | Val |
| 4.1 Were there a reasonable number of participants with the outcome? | | Yes | Yes |
| 4.2 Were continuous and categorical predictors handled appropriately? | | Probably Yes | Probably Yes |
| 4.3 Were all enrolled participants included in the analysis? | | Yes | Yes |
| 4.4 Were participants with missing data handled appropriately? | | Probably Yes | Probably Yes |
| 4.5 Was selection of predictors based on univariable analysis avoided? | | Unclear |  |
| 4.6 Were complexities in the data (e.g., censoring, competing risks, sampling of controls) accounted for appropriately? | | Yes | Yes |
| 4.7 Were relevant model performance measures evaluated appropriately? | | Yes | Yes |
| 4.8 Were model overfitting and optimism in model performance accounted for? | | No |  |
| 4.9 Do predictors and their assigned weights in the final model correspond to the results from multivariable analysis? | | No |  |
| **Risk of bias introduced by the analysis** | **Risk:**  (low/high/unclear) | High | High |
| *Rationale of bias rating:*  Relatively small number of participants. Unclear how many samples per patient were taken. | | | |
| **Overall judgement about risk of bias and applicability of the prediction model evaluation** | | | |
| **Overall judgement of risk of bias** | **Risk:**  (low/high/unclear) | High | |
| *Summary of sources of potential bias:*  Domain 3 and 4. | | | |
| **Overall judgement of applicability** | **Concern:**  (low/high/unclear) | Low | |
| *Summary of applicability concerns:*  Although there is a high risk of bias, this research matches the review question. | | | |

| **Study: Carra G et al. Performance of a prediction model for elevated intracranial pressure in traumatic brain injury: a prospective observational study. ESICM LIVES 2022: part 1. ICMx 10(suppl 2): 225-226** | | | |
| --- | --- | --- | --- |
| **Domain 1: Participants** | | | |
| **A. Risk of Bias** | | | |
| *Describe the sources of data and criteria for participant selection:*    *“Patients with severe TBI and invasive ICP monitoring admitted to the intensive care unit of the University Hospitals Leuven, Belgium, between January 2020 and April 2021, were included in the study.”* | | | |
|  | | Dev | Val |
| 1.1 Were the appropriate data sources used, e.g. cohort, RCT or nested case-control study data? | | N/A | Probably Yes |
| 1.2 Were all inclusions and exclusions of participants appropriate? | | N/A | Probably Yes |
| **Risk of bias introduced by selection of participants:** | **Risk:**  (low/high/unclear) | N/A | Low |
| *Rationale of bias rating:*  The authors appear to include all patients during a certain period in time. | | | |
| **B. Applicability** | | | |
| *Describe included participants, setting and dates:*  *“Patients with severe TBI and invasive ICP monitoring admitted to the intensive care unit of the University Hospitals Leuven, Belgium, between January 2020 and April 2021, were included in the study.”* | | | |
| **Concern that the included participants and setting do not match the review question** | **Concern:**  (low/high/unclear) | N/A | Low |
| *Rationale of applicability rating:*  Participants are TBI patients. | | | |
| **Domain 2: Predictors** |  |  |  |
| **A. Risk of Bias** |  |  |  |
| *List and describe predictors included in the final model, e.g., definition and timing of assessment:*  “*Minute-by-minute ICP and mean arterial blood pressure signals were collected and predictions of harmful ICP doses were computed.”* | | | |
|  | | Dev | Val |
| 2.1 Were predictors defined and assessed in a similar way for all participants? | | N/A | Yes |
| 2.2 Were predictor assessments made without knowledge of outcome data? | | N/A | Yes |
| 2.3 Are all predictors available at the time the model is intended to be used? | | N/A | Yes |
| **Risk of bias introduced by predictors or their assessment** | **Risk:**  (low/high/unclear) | N/A | Low |
| *Rationale of bias rating:*  Predictors were determined in a previous study. | | | |
| **B. Applicability** |  |  |  |
| **Concern that the definition, assessment, or timing of predictors in the model do not match the review questions** | **Concern:**  (low/high/unclear) | N/A | Low |
| *Rationale of applicability rating:*  Used predictors are a reasonable choice. | | | |
| **Domain 3: Outcome** | | | |
| **A. Risk of Bias** | | | |
|  | | Dev | Val |
| 3.1 Was the outcome determined appropriately? | | N/A | Yes |
| 3.2 Was a pre-specified or standard outcome definition used? | | N/A | Yes |
| 3.3 Were predictors excluded from the outcome definition? | | N/A | Yes |
| 3.4 Was the outcome defined and determined in a similar way for all participants? | | N/A | Yes |
| 3.5 Was the outcome determined without knowledge of predictor information? | | N/A | Yes |
| 3.6 Was the time interval between predictor assessment and outcome determination appropriate? | | N/A | Probably Yes |
| **Risk of bias introduced by the outcome or its determination** | **Risk:**  (low/high/unclear) | N/A | Low |
| *Rationale of bias rating:*  Clear outcome definition was used. | | | |
| **B. Applicability** | | | |
| *At what time point was the outcome determined:*  In a previous study.  *If a composite outcome was used, describe the relative frequency/distribution of each contributing outcome:*  N/A. | | | |
| **Concern that the outcome, its definition, timing or determination do not match the review question** | **Concern:**  (low/high/unclear) | N/A | Low |
| Rationale of applicability rating:  Outcome is exactly what this review aims to investigate. | | | |
| **Domain 4: Analysis** | | | |
| **Risk of Bias** | | | |
| *Describe numbers of participants, number of candidate predictors, outcome events and events per candidate predictor:*  14 patients were included. “*Fourteen patients with severe TBI were included in this prospective study.*” | | | |
| *Describe how the model was developed (for example in regards to modelling technique (e.g. survival or logistic modelling), predictor selection, and risk group definition):*  Gaussian Processes was used. “W*e previously developed a machine-learning (ML) model that predicts events of potentially harmful ICP dose 30 min ahead.*” | | | |
| *Describe whether and how the model was validated, either internally (e.g., bootstrapping, cross validation, random split sample) or externally (e.g., temporal validation, geographical validation, different setting, different type of participants):*  This study externally (prospectively) validates a model developed in a previous study, using a different dataset. | | | |
| *Describe the performance measures of the model, e.g., (re)calibration, discrimination, (re)classification, net benefit, and whether they were adjusted for optimism:*  Accuracy, sensitivity, specificity. | | | |
| *Describe any participants who were excluded from the analysis:*  Not mentioned. | | | |
| *Describe missing data on predictors and outcomes as well as methods used for missing data:*  Not mentioned. | | | |
|  | | Dev | Val |
| 4.1 Were there a reasonable number of participants with the outcome? | | N/A | No |
| 4.2 Were continuous and categorical predictors handled appropriately? | | N/A | Probably Yes |
| 4.3 Were all enrolled participants included in the analysis? | | N/A | Yes |
| 4.4 Were participants with missing data handled appropriately? | | N/A | Probably Yes |
| 4.5 Was selection of predictors based on univariable analysis avoided? | | N/A |  |
| 4.6 Were complexities in the data (e.g., censoring, competing risks, sampling of controls) accounted for appropriately? | | N/A | No |
| 4.7 Were relevant model performance measures evaluated appropriately? | | N/A | Yes |
| 4.8 Were model overfitting and optimism in model performance accounted for? | | N/A |  |
| 4.9 Do predictors and their assigned weights in the final model correspond to the results from multivariable analysis? | | N/A |  |
| **Risk of bias introduced by the analysis** | **Risk:**  (low/high/unclear) | N/A | High |
| *Rationale of bias rating:*  Relatively small number of participants, although this is a first-of-its-kind prospective setting. | | | |
| **Overall judgement about risk of bias and applicability of the prediction model evaluation** | | | |
| **Overall judgement of risk of bias** | **Risk:**  (low/high/unclear) | Low | |
| *Summary of sources of potential bias:*  Domain 4. | | | |
| **Overall judgement of applicability** | **Concern:**  (low/high/unclear) | Low | |
| *Summary of applicability concerns:*  Although there is a high risk of bias, this research matches the review question. | | | |

| **Study: Petrov D et al. Prediction of intracranial pressure crises after severe traumatic brain injury using machine learning algorithms. J Neurosurg. 2023 Jan 27; 1-8** | | | |
| --- | --- | --- | --- |
| **Domain 1: Participants** | | | |
| **A. Risk of Bias** | | | |
| *Describe the sources of data and criteria for participant selection:*    “ *All patients admitted to the University of Pennsylvania Health System between April 2015 and January 2019 with severe TBI (GCS score < 8) who underwent intraparenchymal ICP monitor placement and had physiological data continuously recorded and time-synchronized onto a bedside recording unit (CNS Monitor, Moberg ICU Solutions) were retrospectively identified. Patients with severe TBI monitored solely using external ventricular catheters were excluded.*” | | | |
|  | | Dev | Val |
| 1.1 Were the appropriate data sources used, e.g. cohort, RCT or nested case-control study data? | | Yes | Yes |
| 1.2 Were all inclusions and exclusions of participants appropriate? | | Yes | Yes |
| **Risk of bias introduced by selection of participants:** | **Risk:**  (low/high/unclear) | Low | Low |
| *Rationale of bias rating:*  Clear patient selection process. | | | |
| **B. Applicability** | | | |
| *Describe included participants, setting and dates:*  “*All patients admitted to the University of Pennsylvania Health System between April 2015 and January 2019 with severe TBI (GCS score < 8) who underwent intraparenchymal ICP monitor placement and had physiological data continuously recorded and time-synchronized onto a bedside recording unit (CNS Monitor, Moberg ICU Solutions) were retrospectively identified.”* | | | |
| **Concern that the included participants and setting do not match the review question** | **Concern:**  (low/high/unclear) | Low | Low |
| *Rationale of applicability rating:*  Participants are TBI patients. | | | |
| **Domain 2: Predictors** |  |  |  |
| **A. Risk of Bias** |  |  |  |
| *List and describe predictors included in the final model, e.g., definition and timing of assessment:*  *“Continuous ICP data were extracted from each monitoring period (range 4–96 hours of monitoring).”* | | | |
|  | | Dev | Val |
| 2.1 Were predictors defined and assessed in a similar way for all participants? | | Yes | Yes |
| 2.2 Were predictor assessments made without knowledge of outcome data? | | Yes | Yes |
| 2.3 Are all predictors available at the time the model is intended to be used? | | Yes | Yes |
| **Risk of bias introduced by predictors or their assessment** | **Risk:**  (low/high/unclear) | Low | Low |
| *Rationale of bias rating:*  ICP itself is used as predictor, which is not necessarily a problem. | | | |
| **B. Applicability** |  |  |  |
| **Concern that the definition, assessment, or timing of predictors in the model do not match the review questions** | **Concern:**  (low/high/unclear) | Low | Low |
| *Rationale of applicability rating:*  Used predictor is a reasonable choice. | | | |
| **Domain 3: Outcome** | | | |
| **A. Risk of Bias** | | | |
|  | | Dev | Val |
| 3.1 Was the outcome determined appropriately? | | Yes | Yes |
| 3.2 Was a pre-specified or standard outcome definition used? | | Yes | Yes |
| 3.3 Were predictors excluded from the outcome definition? | | Yes | Yes |
| 3.4 Was the outcome defined and determined in a similar way for all participants? | | Yes | Yes |
| 3.5 Was the outcome determined without knowledge of predictor information? | | Yes | Yes |
| 3.6 Was the time interval between predictor assessment and outcome determination appropriate? | | Yes | Yes |
| **Risk of bias introduced by the outcome or its determination** | **Risk:**  (low/high/unclear) | High | High |
| *Rationale of bias rating:*  An ICP threshold was used, but in combination with a rather short duration. | | | |
| **B. Applicability** | | | |
| *At what time point was the outcome determined:*  In the beginning of the study.  *If a composite outcome was used, describe the relative frequency/distribution of each contributing outcome:*  N/A. | | | |
| **Concern that the outcome, its definition, timing or determination do not match the review question** | **Concern:**  (low/high/unclear) | Low | Low |
| Rationale of applicability rating:  Outcome is what this study aims to investigate, although ICP duration is rather short. | | | |
| **Domain 4: Analysis** | | | |
| **Risk of Bias** | | | |
| *Describe numbers of participants, number of candidate predictors, outcome events and events per candidate predictor:*  30 patients were included in de training dataset: the eventual model with the best accuracy score was trained using 2795 events with a 10-minute lead time and 1-hour feature window (656 crisis and 2139 normal events).  5 patients were included in the validation set; no number of events is mentioned. Furthermore, there is 1 patient unaccounted for; possibly used for “tuning” (there were 36 patients in total). | | | |
| *Describe how the model was developed (for example in regards to modelling technique (e.g. survival or logistic modelling), predictor selection, and risk group definition):*  *“Three machine learning algorithms were trained to*  *predict ICP crises: 1) sklearn, random forest; 2) XGBoost,*  *extreme gradient boosting; and 3) sklearn, light gradient*  *boosting model (LGBM). These three algorithms were fit*  *to the labeled data set to create the prediction model.*  *The most predictive algorithm was identified and the*  *model was improved using feature selection and hyperparameter tuning.”* | | | |
| *Describe whether and how the model was validated, either internally (e.g., bootstrapping, cross validation, random split sample) or externally (e.g., temporal validation, geographical validation, different setting, different type of participants):*  This study internally validates the model using 5/36 patients that were not used for model development. | | | |
| *Describe the performance measures of the model, e.g., (re)calibration, discrimination, (re)classification, net benefit, and whether they were adjusted for optimism:*  Accuracy. | | | |
| *Describe any participants who were excluded from the analysis:*  1 patient is not accounted for during the analysis process. | | | |
| *Describe missing data on predictors and outcomes as well as methods used for missing data:*  The authors imputed missing values with mean values. | | | |
|  | | Dev | Val |
| 4.1 Were there a reasonable number of participants with the outcome? | | No | No |
| 4.2 Were continuous and categorical predictors handled appropriately? | | Probably Yes | Probably Yes |
| 4.3 Were all enrolled participants included in the analysis? | | Yes | No |
| 4.4 Were participants with missing data handled appropriately? | | Yes | Yes |
| 4.5 Was selection of predictors based on univariable analysis avoided? | | Unclear |  |
| 4.6 Were complexities in the data (e.g., censoring, competing risks, sampling of controls) accounted for appropriately? | | No | No |
| 4.7 Were relevant model performance measures evaluated appropriately? | | Yes | No |
| 4.8 Were model overfitting and optimism in model performance accounted for? | | Probably Yes |  |
| 4.9 Do predictors and their assigned weights in the final model correspond to the results from multivariable analysis? | | Unclear |  |
| **Risk of bias introduced by the analysis** | **Risk:**  (low/high/unclear) | High | High |
| *Rationale of bias rating:*  Relatively small number of participants. Unclear how many samples per patient were taken. One patient is not accounted for. | | | |
| **Overall judgement about risk of bias and applicability of the prediction model evaluation** | | | |
| **Overall judgement of risk of bias** | **Risk:**  (low/high/unclear) | High | |
| *Summary of sources of potential bias:*  Domain 3 and 4. | | | |
| **Overall judgement of applicability** | **Concern:**  (low/high/unclear) | High | |
| *Summary of applicability concerns:*  This research matches the review question, but one patient is unaccounted for during the analysis. | | | |

| **Study: Carra G et al. Development and External Validation of a Machine Learning Model for the Early Prediction of Doses of Harmful Intracranial Pressure in Patients with Severe Traumatic Brain Injury. J Neurotrauma. 2023 Mar; 40(5-6): 514-522** | | | |
| --- | --- | --- | --- |
| **Domain 1: Participants** | | | |
| **A. Risk of Bias** | | | |
| *Describe the sources of data and criteria for participant selection:*    *“The development cohort included the data of 290 patients from six prospectively and retrospectively collected databases: the Brain-IT is a European multi-center database that contains data of 206 adult patients with severe TBI admitted to 22 intensive care units (ICUs) between March 2003 and July 2005.*  *The external validation cohort was composed of 264 patients included in the High Resolution Sub-Study of the Collaborative European NeuroTrauma Effectiveness Research in Traumatic Brain Injury (CENTER-TBI) dataset.*  *Patients were declared eligible for the study if their ICP recordings were acquired with an intraparenchymal ICP probe.”* | | | |
|  | | Dev | Val |
| 1.1 Were the appropriate data sources used, e.g. cohort, RCT or nested case-control study data? | | Yes | Yes |
| 1.2 Were all inclusions and exclusions of participants appropriate? | | Yes | Yes |
| **Risk of bias introduced by selection of participants:** | **Risk:**  (low/high/unclear) | Low | Low |
| *Rationale of bias rating:*  Patient eligibility appears rational, and various sources are used. | | | |
| **B. Applicability** | | | |
| *Describe included participants, setting and dates:*  *“The development cohort included the data of 290 patients from six prospectively and retrospectively collected databases: the Brain-IT is a European multi-center database that contains data of 206 adult patients with severe TBI admitted to 22 intensive care units (ICUs) between March 2003 and July 2005.*  *The external validation cohort was composed of 264 patients included in the High Resolution Sub-Study of the Collaborative European NeuroTrauma Effectiveness Research in Traumatic Brain Injury (CENTER-TBI) dataset.”* | | | |
| **Concern that the included participants and setting do not match the review question** | **Concern:**  (low/high/unclear) | Low | Low |
| *Rationale of applicability rating:*  Participants are TBI patients. | | | |
| **Domain 2: Predictors** |  |  |  |
| **A. Risk of Bias** |  |  |  |
| *List and describe predictors included in the final model, e.g., definition and timing of assessment:*  “*The ML models were used, with minute-by-minute ICP and mean arterial blood pressure signals as inputs.*” | | | |
|  | | Dev | Val |
| 2.1 Were predictors defined and assessed in a similar way for all participants? | | Yes | Yes |
| 2.2 Were predictor assessments made without knowledge of outcome data? | | Yes | Yes |
| 2.3 Are all predictors available at the time the model is intended to be used? | | Yes | Yes |
| **Risk of bias introduced by predictors or their assessment** | **Risk:**  (low/high/unclear) | Low | Low |
| *Rationale of bias rating:*  Used predictors are a reasonable choice. | | | |
| **B. Applicability** |  |  |  |
| **Concern that the definition, assessment, or timing of predictors in the model do not match the review questions** | **Concern:**  (low/high/unclear) | Low | Low |
| *Rationale of applicability rating:*  Used predictors are a reasonable choice. | | | |
| **Domain 3: Outcome** | | | |
| **A. Risk of Bias** | | | |
|  | | Dev | Val |
| 3.1 Was the outcome determined appropriately? | | Yes | Yes |
| 3.2 Was a pre-specified or standard outcome definition used? | | Yes | Yes |
| 3.3 Were predictors excluded from the outcome definition? | | Yes | Yes |
| 3.4 Was the outcome defined and determined in a similar way for all participants? | | Yes | Yes |
| 3.5 Was the outcome determined without knowledge of predictor information? | | Yes | Yes |
| 3.6 Was the time interval between predictor assessment and outcome determination appropriate? | | Probably Yes | Probably Yes |
| **Risk of bias introduced by the outcome or its determination** | **Risk:**  (low/high/unclear) | Low | Low |
| *Rationale of bias rating:*  Clear outcome definition was used. | | | |
| **B. Applicability** | | | |
| *At what time point was the outcome determined:*  At the start of the study.  *If a composite outcome was used, describe the relative frequency/distribution of each contributing outcome:*  *“Ensembled GP-based models and RF-based model”*; in the end this is not entirely a composite outcome as the final RF model outputs a single probability. | | | |
| **Concern that the outcome, its definition, timing or determination do not match the review question** | **Concern:**  (low/high/unclear) | Low | Low |
| Rationale of applicability rating:  Outcome is exactly what this review aims to investigate. | | | |
| **Domain 4: Analysis** | | | |
| **Risk of Bias** | | | |
| *Describe numbers of participants, number of candidate predictors, outcome events and events per candidate predictor:*  Training data: 290 patients; 20938 4-hour instances.  Validation data: 264 patients; 25261 instances, 8421 events (50%). | | | |
| *Describe how the model was developed (for example in regards to modelling technique (e.g. survival or logistic modelling), predictor selection, and risk group definition):*  *“In a second phase, to minimize the prediction error of the single submodels and provide to the clinicians a unique model output, we developed a Random Forest (RF) classifier that combines the predictions of the models for the red subareas and provides as single output the probability that the patient will experience events of ICP in the red area in the next 30 min.*  *The red subareas were defined according to the visualization curves as follows:*  *ICP >15 mmHg for more than 180 min,*  *ICP >18 mmHg for more than 70 min,*  *ICP>20 mmHg for more than 35 min,*  *ICP >22 mmHg for more than 25 min,*  *ICP >24 mmHg for more than 18 min,*  *ICP >26 mmHg for more than 14 min,*  *ICP>28 mmHg, ICP >30 mmHg, and ICP >34 mmHg for more than 10 min.”* | | | |
| *Describe whether and how the model was validated, either internally (e.g., bootstrapping, cross validation, random split sample) or externally (e.g., temporal validation, geographical validation, different setting, different type of participants):*  This study externally validates a model that is developed using multiple data sources. | | | |
| *Describe the performance measures of the model, e.g., (re)calibration, discrimination, (re)classification, net benefit, and whether they were adjusted for optimism:*  Area Under the Receiver Operator Curve, accuracy, sensitivity, specificity. | | | |
| *Describe any participants who were excluded from the analysis:*  Not mentioned. | | | |
| *Describe missing data on predictors and outcomes as well as methods used for missing data:*  Authors imputed missing data with mean values. | | | |
|  | | Dev | Val |
| 4.1 Were there a reasonable number of participants with the outcome? | | Yes | Yes |
| 4.2 Were continuous and categorical predictors handled appropriately? | | Yes | Yes |
| 4.3 Were all enrolled participants included in the analysis? | | Yes | Yes |
| 4.4 Were participants with missing data handled appropriately? | | Yes | Yes |
| 4.5 Was selection of predictors based on univariable analysis avoided? | | Yes |  |
| 4.6 Were complexities in the data (e.g., censoring, competing risks, sampling of controls) accounted for appropriately? | | No | Yes |
| 4.7 Were relevant model performance measures evaluated appropriately? | | Yes | Yes |
| 4.8 Were model overfitting and optimism in model performance accounted for? | | Yes |  |
| 4.9 Do predictors and their assigned weights in the final model correspond to the results from multivariable analysis? | | Probably Yes |  |
| **Risk of bias introduced by the analysis** | **Risk:**  (low/high/unclear) | Low | Low |
| *Rationale of bias rating:*  Relatively large number of participants.  The ratio of events and non-events in the validation dataset is 50%. | | | |
| **Overall judgement about risk of bias and applicability of the prediction model evaluation** | | | |
| **Overall judgement of risk of bias** | **Risk:**  (low/high/unclear) | Low | |
| *Summary of sources of potential bias:*  None. | | | |
| **Overall judgement of applicability** | **Concern:**  (low/high/unclear) | Low | |
| *Summary of applicability concerns:*  Low risk of bias, and this research matches the review question. | | | |
